# Colorectal microenvironment determines the prognosis of colorectal cancer

**DOI:** 10.1038/s12276-025-01599-7

**Published:** 2026-01-07

**Authors:** Yeong Hak Bang, Ji Hye Choi, Kyunghee Park, Boram Lee, Kyung Yeon Han, Dae Hee Pyo, Yong Beom Cho, Tae-You Kim, Kyu Joo Park, Seung-Bum Ryoo, Sung-Bum Kang, Chang Sik Yu, Jaeim Lee, Kil-yong Lee, Kyu-Tae Kim, Jin-Young Lee, Hoang Bao Khanh Chu, Nameeta Shah, Shashank Gupta, Pranali Sonpatki, Young-Joon Kim, Woong-Yang Park

**Affiliations:** 1 Samsung Genome Institute, Samsung Medical Center, Seoul, Republic of Korea; 2 Department of Oncology, Asan Medical Center, University of Ulsan College of Medicine, Seoul, Republic of Korea; 3 Department of Health Sciences and Technology, Samsung Advanced Institute of Health Sciences and Technology, Sungkyunkwan University, Seoul, Republic of Korea; 4 Department of Pathology and Translational Genomics, Samsung Medical Center, Sungkyunkwan University School of Medicine, Seoul, Republic of Korea; 5 Department of Surgery, Eunpyeong St. Mary’s Hospital, College of Medicine, The Catholic University of Korea, Seoul, Republic of Korea; 6 Department of Surgery, Samsung Medical Center, Sungkyunkwan University School of Medicine, Seoul, Republic of Korea; 7 Division of Hematooncology, Department of Internal Medicine, Seoul National University Hospital, Seoul National University College of Medicine, Seoul, Republic of Korea; 8 Cancer Research Institute, Seoul National University College of Medicine, Seoul, Republic of Korea; 9 IMBdx Inc., Seoul, Republic of Korea; 10 Department of Molecular Medicine and Biopharmaceutical Sciences, Graduate School of Convergence Science and Technology, Seoul National University, Seoul, Republic of Korea; 11 Department of Surgery, Seoul National University Hospital, Seoul National University College of Medicine, Seoul, Republic of Korea; 12 Department of Surgery, Seoul National University College of Medicine, Seoul National University Bundang Hospital, Seongnam, Republic of Korea; 13 Department of Colon and Rectal Surgery, Asan Medical Center, University of Ulsan College of Medicine, Seoul, Republic of Korea; 14 Department of Surgery, Uijeongbu St. Mary’s Hospital, College of Medicine, The Catholic University of Korea, Seoul, Republic of Korea; 15 Department of Physiology, Ajou University School of Medicine, Suwon, Republic of Korea; 16 Department of Biochemistry, College of Life Science and Biotechnology, Yonsei University, Seoul, Republic of Korea; 17 Amaranth Medical Analytics, Bengaluru, India; 18 LepiDyne Co. Ltd., Seoul, Republic of Korea; 19 Geninus, Seoul, Republic of Korea

**Keywords:** Prognostic markers, Tumour biomarkers, Gastrointestinal cancer

## Abstract

Here we aimed to evaluate the feasibility of distinguishing colorectal microenvironments that support cancer cell growth from those that do not. We hypothesized that patients whose non-tumor-bearing tissue (NBT) obtained from the furthest margins of resected cancer specimens resembled the tumor had a poorer prognosis. Patients with colorectal cancer were divided into groups with tumor-supportive (TSM) or healthy microenvironments using bulk RNA sequencing data from 273 paired NBT and tumor samples. Patients in the TSM group exhibited significantly poorer 5-year recurrence-free survival and overall survival compared with those in the healthy microenvironment group. Pathway and 16S rRNA sequencing analyses revealed that NBT and tumors from the TSM group shared a microbiome composition, along with decreased pathway activity related to microvilli maintenance and flavonoid or vitamin metabolic processes. Single-cell RNA sequencing uncovered upregulated interactions between *IL1B*^high^ neutrophils and *OLFM4*^+^ epithelial cells in NBTs from the TSM group, as well as organized microniches in TSM tumors, featuring interactions between *EMP1*^high^ epithelial cells, *IL1B*^high^ neutrophils and *GZMK*^high^ CD8^+^ T cells. Collectively, the colorectal microenvironment can serve as a prognostic biomarker to effectively predict cancer invasiveness and tumor-promoting inflammation. Maintaining a healthy colorectal mucosal microenvironment, potentially through dietary intervention, is crucial.

## Colorectal microenvironments predict cancer prognosis and survival

Colorectal cancer (CRC) is a complex disease with varied genetic and environmental factors. Current genomic markers help to predict treatment outcomes for advanced cases but not for early-stage CRC. About 30–40% of CRC cases return after surgery, indicating a need for better predictive tools. Researchers explored using normal-looking tissue near tumors as a potential marker for recurrence. This study involved 273 patients with stage II or III CRC who underwent surgery. Researchers used RNA sequencing to analyze both the tumor and the nearby normal tissues. They identified specific genes that were more active in tumors and used these to classify patients into two groups: those with a tumor-supportive environment and those with a healthier environment. The results showed that patients with tumor-like features in their normal tissue had worse survival rates. This suggests that the surrounding tissue’s condition can predict cancer recurrence.

This summary was initially drafted using artificial intelligence, then revised and fact-checked by the author.

## Introduction

Colorectal cancer (CRC) is highly heterogeneous at the genomic and transcriptomic levels. Genomic biomarkers, namely microsatellite instability (MSI) and extended *RAS* and *BRAF* mutational status, are routinely used for prognostication and treatment prediction for metastatic settings in clinical practice^1–4^. However, their use is not recommended in adjuvant settings, except for MSI, owing to their lack of predictive value for treatment benefits^3–6^. Overall, 30–40% of surgically resected CRC cases recur, leading to a poor prognosis^7^. Thus, there is an unmet need for predictive biomarkers for CRC recurrence.

Previous studies have focused on tumor mutational profiles or microenvironments as potential biomarkers. However, the heterogeneous molecular characteristics and microbiota within tumors present a substantial impediment in facilitating comparative analyses across patients^8–11^. To bypass intratumoral heterogeneity, we explored the potential of using histologically normal tissue (non-tumor-bearing tissue (NBT)) obtained from the furthest margins of resected specimens as prognostic biomarkers. While NBTs are commonly used as a normal control in cancer studies, growing evidence suggests that cumulative genetic alterations during carcinogenesis leave NBT in an intermediate, preneoplastic state characterized by morphologically normal but molecularly altered cells^12^. In this context, NBT exhibits molecular features that are intermediate between tumors and normal tissues from healthy controls, highlighting its potential as a hallmark of tumorigenesis or tumor progression^12–16^.

In the case of CRC, the disruption of the protective mucus layer, along with increased interactions within dysbiotic microbial networks, represents a critical step in CRC development^17–19^. Therefore, NBTs may serve as an indicator of local tumor recurrence. In addition, disrupted NBT may be correlated with metachronous recurrence. Bacteria-infected cancer cells were reported to be related to colon cancer metastasis^8^, and bacterial biofilms—aggregations of the microbial community that contact unshielded epithelial cells and invade the tumor—have been observed not only within the tumor but also in NBTs, even far from the tumor^18,20,21^.

We hypothesized that distinct colorectal microenvironments either support or inhibit the growth of cancer cells and that the NBT reflects these microenvironments. In addition, we assumed that the more closely the characteristics of the NBT resemble those of the tumor, the greater the likelihood of supporting cancer cell progression and promoting disease recurrence. Here, as proof of concept, we used bulk RNA sequencing (RNA-seq) to classify patients into two subgroups—those with a tumor-supportive microenvironment (TSM) and those with a healthy microenvironment (HM)—and investigated their clinical outcomes. In addition, we analyzed multi-omics data, including 16S ribosomal RNA-seq and single-cell RNA-seq (scRNA-seq) of NBT and tumor tissues to compare their biological characteristics between the patient groups.

## Material and methods

### Patient selection and recruitment

**Fig. 1:**
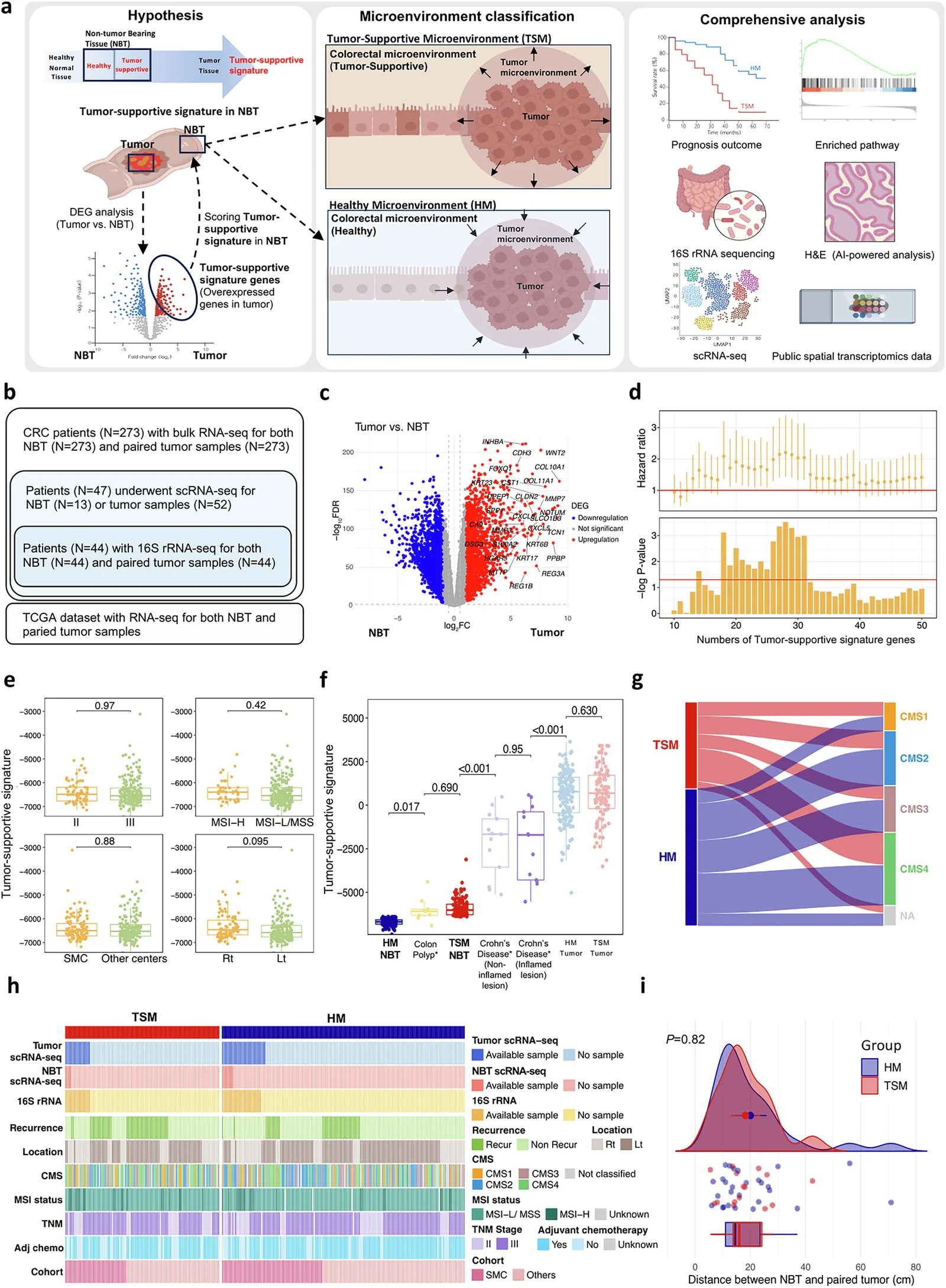
Study outline and selection process of tumor-supportive signature genes. **a** Study approach. We first investigated the DEGs between tumors and NBT and designated overexpressed genes in tumors as tumor-supportive signature genes. We categorized the subgroups on the basis of the profile of tumor-supportive signature genes in NBTs (relatively high tumor-supportive signature scores in NBT as TSM; relatively low scores as HM). Our analysis incorporated multi-omics data—encompassing bulk RNA-seq data, H&E staining data of NBT, 16S rRNA-seq data and scRNA-seq data—and publicly available spatial transcriptomics data. **b** Summary of cohorts: bulk RNA-seq analysis was conducted on NBT and paired tumor samples from 273 patients. Among these, NBT and tumor samples from 47 patients were also subjected to scRNA-seq analysis. In addition, NBT and paired tumor samples from 44 patients were available for 16S rRNA-seq. The bulk RNA-seq data from TCGA, which included colorectal and other cancer types, were used for validation. **c** Volcano plot illustrating the DEGs between tumors and NBT. Red and blue dots represent overexpressed and underexpressed genes in tumors, respectively. Tumor-supportive signature genes are labeled. **d** Simulation results of the prognostic difference (Cox HR) between the TSM and HM groups. The *x* axis indicates the number of DEGs (mostly overexpressed in tumors) used for calculation, and the *y* axis indicates the HR (top) and −log *P* value (bottom) between the two groups (TSM versus HM). **e** Comparison of tumor-supportive signature scores by cohort characteristics with TNM stage, MSI status, medical center and tumor sidedness. **f** Comparison of tumor-supportive signature between NBT from the HM and TSM groups, colon polyp tissue, colon tissue from Crohn’s disease (both noninflamed and inflamed lesions) and tumor tissue from the HM and TSM groups. ^*^Publicly available data for colon polyp tissue and Crohn’s disease (GSE208303). **g** CMSs according to the microenvironment-based classification (TSM and HM). (**h**) Clinical metadata of the study samples, including information on scRNA-seq analysis, 16S rRNA-seq analysis, disease recurrence, tumor location, CMSs, MSI status, TNM stage, adjuvant chemotherapy and medical center. **i** Comparison of the distance between paired NBT and tumor samples for the TSM and HM groups. The density plot shows the distribution of distances, while the scatter plot and box plot below further illustrate individual data points and summary statistics. DEGs, differentially expressed genes; NBT, non-tumor-bearing tissue; TSM, tumor-supportive microenvironment; HM, healthy microenvironment; H&E, hematoxylin and eosin staining; RNA-seq, RNA sequencing; scRNA-seq, single-cell RNA sequencing; 16S rRNA-seq, 16S rRNA sequencing; CRC, colorectal cancer; TCGA, The Cancer Genome Atlas; Rt, right-sided; Lt, left-sided; CMS, consensus molecular subtype; MSI, microsatellite instability; MSI-L, microsatellite instability–low; MSS, microsatellite stable; MSI-H, microsatellite instability–high; Adj chemo, adjuvant chemotherapy.

We investigated paired chemotherapy-naive tumors and NBTs obtained from the furthest margins of resected specimens from 273 patients with stage II or III CRC who underwent R0 surgical resection at the Samsung Medical Center, Seoul National University Hospital, Bundang Seoul National University Hospital, Asan Medical Center or Uijeongbu St. Mary’s Hospital (Republic of Korea) between July 2009 and July 2019 (Fig. 1a,b and Supplementary Fig. 1a). This study was conducted in accordance with the principles of the Declaration of Helsinki and was approved by the Institutional Review Board of each institution (document ID nos. 2017-01-131, 2103-121-1206, B-1709-423-306, 2017-1350 and XC17TNDI0068, respectively). This study was also approved by the Institutional Review Board of Yonsei University (document ID no. 7001988-201910-BR-727-02).

### Patient survival analysis

Recurrence-free survival (RFS) was calculated from the date of surgery to the date of the first tumor recurrence or mortality from any cause, whichever occurred first. Overall survival (OS) was defined as the time from the date of surgery to the date of death from any cause or the last follow-up. The Kaplan–Meier method was used to estimate survival outcomes, and the log-rank test was used to compare survival outcomes among subgroups. Univariate and multivariate analyses of RFS and OS were performed using Cox proportional hazard models. Variables with a potential relationship (*P* < 0.1) in the univariate analysis were included in the multivariate analysis. A *P* value <0.05 was considered statistically significant. All statistical analyses were performed using R software (version 4.0.5; R Foundation for Statistical Computing).

### RNA extraction and sequencing of primary tissue samples

NBTs and matched tumor fresh–frozen tissues (20–40 mg) obtained from patients with CRC were dissected and homogenized three to four times for 15 s at a frequency of 30 Hz using a Tissue Lyser II (QIAGEN). RNA was extracted using the RNeasy Mini Kit (QIAGEN) according to the standard tissue RNA extraction protocol. Total RNA concentration was calculated using Quant-IT RiboGreen (R11490; Invitrogen). To assess the integrity of the total RNA, the samples were evaluated using a TapeStation RNA screentape (5067–5576; Agilent). Only high-quality RNA preparations (RNA integrity number >7.0) were used for the RNA library construction. The total RNA was subjected to rRNA depletion using the Ribo-Zero Gold rRNA Removal Kit (MRZG12324; Illumina). Subsequently, 2 μl of a 100-fold diluted external RNA controls consortium (ERCC) Mix2 solution of the ERCC RNA Spike-In Mix (4456740; Ambion) was added. An RNA-seq library was prepared using a TruSeq RNA Sample Prep Kit (Illumina). The libraries were then subjected to an Illumina HiSeq2000 platform (Illumina), and paired-end (2 × 100 base pairs (bp)) sequencing was performed by Macrogen.

### RNA-seq data preprocessing

The human GRCh38 reference genome and gene annotation GTF file (GENCODE version 27) were obtained from GENCODE (https://www.gencodegenes.org/human/), and the ERCC sequences’ FASTA and GTF annotations were obtained from Thermo Fisher Scientific (https://assets.thermofisher.com/TFS-Assets/LSG/manuals/ERCC92.zip). Genome indexing and alignment were performed using STAR (version 2.5.3a), and gene expression levels were quantified using RSEM (version 1.3.0)^22^.

### Tumor-supportive signature gene selection and classification of the patients

Differentially expressed genes (DEGs) were identified using edgeR software (version 3.38.4)^23^. Count matrices and genes were generated using the DGEList and filterByExpr functions. Principal component analysis was performed to confirm that tumor and NBT samples were well separated (Supplementary Fig. 1b). The trimmed mean of M-values normalization was performed using calcNormFactors. Finally, the negative binomial dispersion parameters were estimated using estimateDisp. We obtained DEGs by running glmQLFit and glmQLFTest and filtered them using log_2_(fold change (FC)) >1, false discovery rate (FDR) <0.05 and log_2_(CPM (counts per million)) >3 thresholds. Among the DEGs, we selected the top 28 enriched genes in tumors as tumor-supportive signature genes. To quantify the tumor-supportive signature score, we performed single-sample gene set enrichment analysis (ssGSEA)^24^ using the tumor-supportive signature genes for each sample. CPM were generated using edgeR^23^ with log = T. ssGSEA was performed using the gsva function in the GSVA package with the options mx.diff = F, kcdf = ‘Poisson’, method = ‘ssgsea’ and ssgsea.norm = F. Patients were divided into the TSM or HM groups on the basis of whether their NBT tumor-supportive signature score was higher or lower than that of the mean value of the study population (mean ssGSEA score, −6,387), respectively. For validation, we applied the same tumor-supportive signature score criteria to a colon dataset from The Cancer Genome Atlas (TCGA). For the TCGA pancancer analysis, tumor-supportive signature genes were identified separately for each cancer type by comparing paired NBTs and tumors. We then divided the subgroups using the same classification method applied in CRC.

### Application of tumor-supportive signature to Crohn’s disease and colon polyp tissues

We obtained and analyzed public bulk RNA-seq data from GSE208303. We calculated the scores using the same method applied to assess the CRC study population.

### DEGs and related pathway analysis

Gene set enrichment analysis (GSEA)^25^ was performed using gseGO in the clusterProfiler package (version 4.4.4). gseGO was performed using the options keyType = ‘SYMBOL’, pvalueCutoff = 0.05, OrgDb = org.Hs.eg.db and pAdjustMethod = ‘fdr’.

### CMS prediction

The consensus molecular subtype (CMS) was predicted using the CMScaller package (version 0.99.2)^26^.

### Inference of immune cell composition using bulk RNA-seq data

The composition of immune cells was assessed using CIBERSORTx^27^, which involved analyzing the RNA-seq transcripts per million matrix of the cohort. The analysis was conducted in absolute mode, using the LM22 gene set. Quantile normalization was disabled during the analysis, and 1,000 permutations were performed to ensure robustness. In addition, batch correction using the B mode was applied to address any batch differences between the RNA-seq data in this study and the LM22 signature, which was originally derived from microarray data.

### Crypt segmentation identification model

Data preparation involved using crypt annotations on 15 whole slide images, which were resized to 20× magnification. The regions of interest were extracted and tiled into 256 × 256 pixel patches, yielding a total of 4,987 patches. These were then split into training and testing sets with a 60:40 ratio. One image was from the GTEX portal (https://gtexportal.org/home/) and 14 from Amaranth Medical Analytics. Both models utilize a U-Net architecture with a ResNet-50 encoder, using ResNet bottleneck layers for effective downsampling and a ConvBlock bridge for enhanced feature transformation. The progressive upsampling mechanism was reinforced by skip connections, ensuring pixel-level accuracy in segmentation tasks. We used a range of data augmentations to increase model robustness and trained both models using dice loss as the primary optimization objective, with a learning rate of 0.001. Each model was trained for 1000 epochs to achieve optimal segmentation performance. For model selection, we chose the epoch with the lowest test loss, resulting in optimal performance at epoch 215 for colon tissue segmentation and epoch 747 for crypt segmentation, as overfitting was observed beyond these points. To enhance the model robustness and reflect real-world variability, we applied a diverse set of training-time augmentations. These included staining augmentations to account for staining variation across different scanning conditions and scanners, geometric transformations, noise-based augmentations and color-based augmentations, aiming to improve model generalizability across variable sample conditions. The sum of the areas within the red lines in relation to the whole slide images was used for correlation analysis with the tumor-supportive signature.

### Single-cell preparation for sequencing

For scRNA-seq, tissue dissociation was performed using a Tumor Dissociation Kit (Miltenyi Biotech) according to the manufacturer’s instructions. In brief, tissues were cut into pieces of 2–4 mm in size and transferred to a C tube containing an enzyme mix (enzymes H, R and A in Roswell Park Memorial Institute (RPMI) 1640 medium). GentleMACS programs (h_tumor_01, h_tumor_02 and h_tumor_02) were run in a MACSmix tube rotator (Miltenyi Biotech) with two 30-min incubation periods at 37 °C between each run. The digested samples were filtered through a 70-μm strainer and washed with RPMI 1640 medium. Each cell suspension purified using a Ficoll-Paque PLUS (GE Healthcare) was processed with 10x Chromium Single Cell 3′ Reagent Kits v3 (10x Genomics) according to the manufacturer’s protocol.

### scRNA-seq and data processing

The scRNA-seq libraries using the 10x Single Cell 3’ v2 Reagent Kit were prepared according to the manufacturer’s protocol (10x Genomics). Sequencing libraries were sequenced on an Illumina HiSeq 4000 platform, targeting 100,000 reads per cell, according to the manufacturer’s instructions (Illumina). Both reads were aligned to the GRCh38 human genome reference sequence and quantified using the CellRanger^28^ software (version 7.0.1). SoupX (version 1.5.2)^29^ and DoubletFinder (version 2.0.3)^30^ were used to remove ambient RNA and doublets. Gene expression was analyzed using the Seurat^31^ software (version 4.0.5). In total, 142,239 cells were considered on the basis of the following criteria: >200 detected genes, <7,000 detected genes and <25% mitochondrial content, with the additional removal of low-quality clusters. The data were normalized using a log-normalized function with a scale factor of 10,000. Variable features were identified using the FindVariableFeatures function, returning 3,000 features. Subsequently, principal component analysis was performed on the basis of a processed expression matrix containing highly variable genes. Subsequently, we applied the Harmony^32^ batch correction package to each sample ID to adjust for potential batch-derived effects across the samples. Uniform manifold approximation and projection was used to visualize the cells in two-dimensional space, followed by the FindNeighbors and FindClusters functions of Seurat. Major cell types were annotated by comparing canonical marker genes and DEGs for each cluster using FindAllMarkers with the Wilcoxon rank-sum test. To compare the abundance of the identified cell types between the TSM and HM groups, we used the scCODA (version 0.1.9) algorithm^33^ implemented in Python (v3.8). Plasmacytoid dendritic cells (DCs) were used as the reference cell type by reference_cell_type = ‘auto’ with an FDR threshold of 0.1. Among these, we selected significant differential composition cell types with an absolute log_2_FC >0.5.

### scRNA-seq analysis of NBTs

We identified a total of 32 cell types, including 16 immune cell and 16 nonimmune cell subsets annotated with canonical markers and DEGs. Myeloid clusters were divided into six clusters, as follows. Neutrophils were characterized by expressing *G0S2*, *CSF3R*^34^ and *IL1B*. The macrophage subsets featured the canonical markers *C1QA, C1QB* and *CD68*^35^, and among these macrophage subgroups, one was distinguished by the expression of *MMP9*. Other myeloid cell subtypes were defined on the basis of canonical DC markers (conventional type 1 DC, *CLEC9A, XCR1* and *CADM1*^35^; conventional type 2 DC, *FCER1A, CD1C* and *CD1E*^35^) and mast cell markers (*KIT, TPSAB1* and *CPA3*)^36^. Unsupervised clustering analysis of T and natural killer (NK) cells identified eight subtypes. Naive-like T cells expressed *CCR7, SELL* and *LEF1*^37^. CD4^+^ T cells were defined on the basis of canonical markers (Th17, *CCR6, KLRB1* and *RORA*; regulatory T cells, *FOXP3, CTLA* and *IL2RA*)^38^. In addition, central memory CD4^+^ T cells were featured on the basis of the expression of *CD69, IL7R* and *GPR183*^39^*. GZMK*^high^ CD8^+^ T cells were characterized by a high expression of *GZMK*, along with *GZMA, GZMB, GZMH* and *PRF1* but without *ENTPD1* expression^40^. The resident-tissue memory CD8^+^ T cells were identified using the canonical markers *ITGAE* (*CD103*) and *CD69*^40^. γδ T cells were characterized on the basis of *TRGC2* and *TRDC* expression^38^, and NK cells expressed *NCAM1*, *FCGR3A* and *KLRF1*^38^. B and plasma cells were identified on the basis of *CD79A* expression along with *CD19* and *MZB1* expression, respectively. Epithelial cells were identified on the basis of *EPCAM* expression and divided into six subsets: colonocyte, *OLFM4*^+^ colonocyte, *PLCG2*^+^ colonocyte, *BEST4*^+^ colonocyte, goblet cell (*FCGBP* and *MUC2*^41^) and enterochromaffin cell (*CHGA*, *CHGB* and *TPH1*^41^). Stromal or endothelial cells (ECs) were identified using fibroblast (*COL3A1* and *THY1*^38^) and EC markers (*ENG* and *PECAM1*^38^). In addition, we further divided the fibroblasts on the basis of DEGs (*ADAMDEC1*^+^ stromal and *SFRP2*^+^ stromal). ECs were identified with canonical markers (telocyte, *SOX6* and *F3*^42^; stalk-like EC, *VWF, ACKR1* and *CD36*^43^; lymphatic EC, *LYVE1* and *PROX1*; pericyte, *NDUFA4L2* and *RGS5*^41^; smooth muscle cell, *CNN1* and *DES*; glial cell, *S100B* and *PLP1*^38,43^).

### scRNA-seq analysis of tumors

The macrophage subsets featured the canonical markers (*C1QA, C1QB* and *CD68*^35^) and were subsequently divided on the basis of the DEGs (*C1QC, SPP1* and *MKI67*). Plasmacytoid and lymphocyte DCs were defined on the basis of *LILRA4, GZMB* plus *IL3RA*^36^ and *LAMP3, CCR7* plus *FSCN1*^41^, respectively. Other DCs, monocytes and neutrophil subgroups were identified using the same canonical markers as used in the NBT analysis. Unsupervised clustering analysis of T and NK cells identified 13 subtypes, including exhausted T cluster (highest expression of exhaustion-related markers, *PDCD1*, *TIGIT* and *LAG3*^39^), follicular helper T cells (*MAF* and *CXCL13*), mucosal-associated invariant T (*SLC4A10* and *TRAV1-*2^39^), stress response T (*BAG3*, and *HSPA1A*^39^), CD4^+^ cytotoxic T (*GZMA, GNLY, PRF1* and *GZMK*^39^) and *MKI67*^+^ T cells (*MKI67*, *PCNA* and *STMN1*^44^). *GZMK*^high^ CD8^+^ T cells, resident-tissue memory CD8^+^ T cells, γδ T cells, regulatory T cells, Th17, naive-like T and NK cells were identified using the same canonical markers as in NBT analysis. Subclusters of stromal cells or ECs were identified using the same canonical markers as those used in NBT. Cancer-associated fibroblasts (CAFs), marked by *FAP*, were further classified into subtypes on the basis of additional canonical markers: inflammatory CAF (*CFD* and *CXCL1*^44^) and myofibroblast-like CAF (*MMP11* and *HOPX*^44^). Epithelial cells were identified with *EPCAM* expression and divided into four subsets: *EMP1*^high^ epithelial cells, *LGR5*^+^ epithelial cells, *MKI67*^+^ epithelial cells and *MUC2*^+^ epithelial cells^41^. We identified each epithelial cell cluster trait using Seurat’s AddModuleScore, which calculates the average expression level of each cell cluster using selected MSigDB hallmark gene sets. A heat map was generated using the pheatmap (version 1.0.12) function with scale = ‘row’. In addition, to identify the *EMP1*^high^ epithelial cell cluster characteristics, we identified DEGs using the FindAllMarkers function in the Seurat R package. The DEG lists were filtered on the basis of the following criteria: expression in ≥20% of the cluster cells, average expression log_2_FC >0.5, and q-value <0.05. Then, we conducted Enrichr^45^ (version 3.2) with the ‘KEGG_2021_Human’ database^46^ for over-representation analysis. CopyKAT v1.0.8^47^ was used to infer copy number profiles and assign with or without copy number alteration labels to each cell. Preprocessed scRNA-seq counts were given as input with default parameters.

### Pseudotime reconstruction and trajectory inference

We estimated single-cell trajectories using Monocle2 (version 2.20.0)^48^. The gene–barcode matrix was normalized using the EstimateSizeFactors function, and the variance of each gene was estimated using the EstimateDispersions function. We used DEGs identified through Seurat’s FindAllMarkers to sort cells. Subsequently, the dimension was reduced using DDRTree and cells were sorted according to pseudotime through the orderCells function.

### Cell–cell communication analysis

CellChat (version 1.6.1)^49^ was used to assess cell–cell communication via interaction network analysis. The TSM and HM group data were processed separately, and each Seurat object was used as an input for CellChat, following the standard protocol (https://github.com/sqjin/CellChat). Population size was considered in the computeCommunProb function with the option population.size = T. Cell–cell communication networks were calculated using the getMaxWeight function, and circle plots were generated using netVisual_aggregate.

### Correlation analysis

To mitigate the potential confounding effects of global cell population abundance differences, we calculated the proportion of each cell type within its respective global population (for example, *EMP1*^high^ epithelial cells within the epithelial cell population, *IL1B*^high^ neutrophils within the myeloid cell population and *GZMK*^high^ CD8^+^ T cells within the T cell population). Subsequently, we performed Pearson correlation analysis to assess the relationships between these cell type clusters.

### Spatial transcriptomics data analysis

The processed sequencing data were obtained from GSE226997. The cell2location (version 0.1.3) tool^50^ was used to map the spatial distribution of the cell types by integrating scRNA-seq and spatial transcriptomic data from a given tissue.

### DNA isolation and 16S rRNA-seq

In total, 44 paired NBT and tumor samples obtained from the same individuals with CRC who underwent resection surgery were used for 16S rRNA analysis. V3–V4 amplicon sequencing data for 16S rRNA were obtained using the Illumina MiSeq Reagent Kit v3 (2 × 300 bp, Illumina). Polymerase chain reaction (PCR) primers (forward, CCTACGGGNGGCWGCAG; reverse, GACTACHVGGGTATCTAATCC) were designed on the basis of the hypervariable regions (V3–V4) of the 16S rRNA. PCR was conducted using 2× KAPA HiFi HotStart ReadyMix (Roche) under the following conditions: 95 °C solution chain for 3 min, 25 cycles of 95 °C for 30 s, 55 °C for 30 s and 72 °C for 45 s, followed by a 72 °C extension for 5 min. Sequencing libraries were then constructed using a TruSeq DNA PCR-Free Sample Preparation Kit (Illumina) and TruSeq Nextera XT index primer (Illumina), as well as 2× KAPA HiFi HotStart ReadyMix (Roche), using the PCR products after purification. Subsequently, paired-end reads were generated by sequencing using a MiSeq platform after determining the quality of the library using a Tapestation 4200 platform (Agilent Technologies) and a Qubit Fluorometer (Thermo Fisher Scientific).

### 16S rRNA-seq analysis

To enhance the sensitivity of estimating the abundance of microbiota in each patient, we first sought to mitigate sequencing bias. We removed contaminated human reads and adapter sequences from the 16S rRNA reads using the Trimmomatic software (version 0.36)^51^. Kraken2 (version 2.12)^52^ was used to detect microbial reads and assign taxonomic classifications using default settings. We then computed the abundance of taxa at the genus level using Braken (version 2.6.2)^53^ with default settings. At the genus level, the relative abundance was calculated as the read count of a specific genus divided by the total number of read genera in each sample. Microbiota beta diversity was calculated using the Bray–Curtis dissimilarity and analyzed by permutational multivariate analysis of variance (PERMANOVA) using the vegan^54^ package (version 2.6). Berger–Parker calculated the microbiota alpha diversity of individual bacterial families with alpha_diversity.py from KrakenTools (version 1.2)^55^, which was analyzed between paired tumor tissue and NBT from the TSM and HM groups using a Wilcoxon signed-rank test.

### RNAscope in situ hybridization

RNAscope in situ hybridization for messenger RNA expression was performed on paired NBT and tumor tissues using an RNAscope 2.5 HD Reagent Brown Kit (catalog no. 322370; Advanced Cell Diagnostics) according to the manufacturer’s instructions.

In brief, 3-mm-thick sections were cut from formalin-fixed, paraffin-embedded tissue samples, and the RNAscope Probe-EB-16S-rRNA (catalog no. 464461; Advanced Cell Diagnostics) was applied.

## Results

### Selection process of tumor-supportive signature genes and their characteristics

We hypothesized that the more similar the NBT was to the tumor, the higher the likelihood of recurrence. As proof of concept, we classified patients into subgroups using bulk RNA-seq data derived from 273 paired NBTs and tumors from surgical specimens of stage II or III CRC (according to the eighth edition of the American Joint Committee on Cancer staging system^7^). To quantify this similarity, we first investigated the DEGs between NBT and tumors of the 273 patients and designated overexpressed genes in tumors as tumor-supportive signature genes (Fig. 1a, b). Patients were classified as having a TSM or an HM if they showed relatively high or low tumor-supportive signatures in NBTs, respectively (Fig. 1a).

Initially, we applied a scoring system using the top 10–50 DEGs with high FC values in tumors (Fig. 1c, d). We scored the expression of the tumor-supportive signature genes in the NBTs using ssGSEA^24^. We evaluated the mean score derived from the top 10–50 DEGs in the NBTs to categorize the patients into two groups that most effectively facilitated prognostic differentiation. Following this, we categorized the patient subgroups using tumor-supportive signature scores from the top 28 DEGs (tumor-supportive signature genes) that exhibited the most significant prognostic differentiation (Fig. 1d and Supplementary Table 1). These tumor-supportive signature genes were associated with maintaining epithelial barrier integrity (*CDH3, CLDN2, COL10A1, DSG3, KRT17, KRT23, KRT6B, REG1B* and *REG3A*), degradation of extracellular matrix components (*MMP3* and *MMP7*), neutrophil chemotaxis (*CXCL5, CXCL8, PPBP* and *SPP1*), growth factors (*INHBA, NOTUM, WNT2* and *FOXQ1*) and cellular environment regulation, including pH balance, transport and cellular response to change in the extracellular environment (*CA9, SLCO1B3* and *TCN1*) (Supplementary Table 1).

The tumor-supportive signature score showed no significant difference according to TNM stage (American Joint Committee on Cancer AJCC 8th edition), microsattelite instability (MSI) status, medical center or tumor sidedness (Fig. 1e). Interestingly, when compared with public bulk RNA-seq data of Crohn’s disease and colon polyps (GSE208303)^56^, the tumor-supportive signature scores of the NBT from the TSM group were similar (*P* = 0.690) to those in the colon polyps dataset but significantly lower (*P* = 0.017) in the NBT of the HM group. The scores from the Crohn’s disease dataset showed intermediate levels between NBT and tumors, regardless of the presence of inflammatory or noninflammatory lesions. No significant difference in the tumor-supportive signature scores between tumors of the TSM and HM groups was observed (*P* = 0.630) (Fig. 1f).

### Baseline characteristics of the study cohort

Approximately two-thirds had left-sided CRC (*n* = 182, 66.7%), while almost three-quarters were diagnosed with stage III CRC (*n* = 204, 74.7%). Most patients exhibited a microsatellite stable phenotype (*n* = 220, 80.6%), and a significant proportion of patients underwent adjuvant treatment (*n* = 207, 75.8%). In this study, no patients had a history of inflammatory bowel disease. In total, 106 (38.8%) patients were classified into the TSM group. No significant differences were observed in the baseline characteristics, including age, sex, TNM stage, tumor sidedness, proportion of patients who underwent adjuvant chemotherapy and CMSs^57^, between the subgroups (Fig. 1g, h and Supplementary Table 2). We measured the distance between tumors and paired NBTs in 42 available surgical specimens (median distance, 15.3 cm; range, 5.5–71.0 cm), which was not associated with the microenvironment-based classification (*ρ* = −0.15, *P* = 0.344) (Fig. 1i and Supplementary Fig. 1c, d).

### Prognostic outcomes of the patients according to subgroup classification

**Fig. 2:**
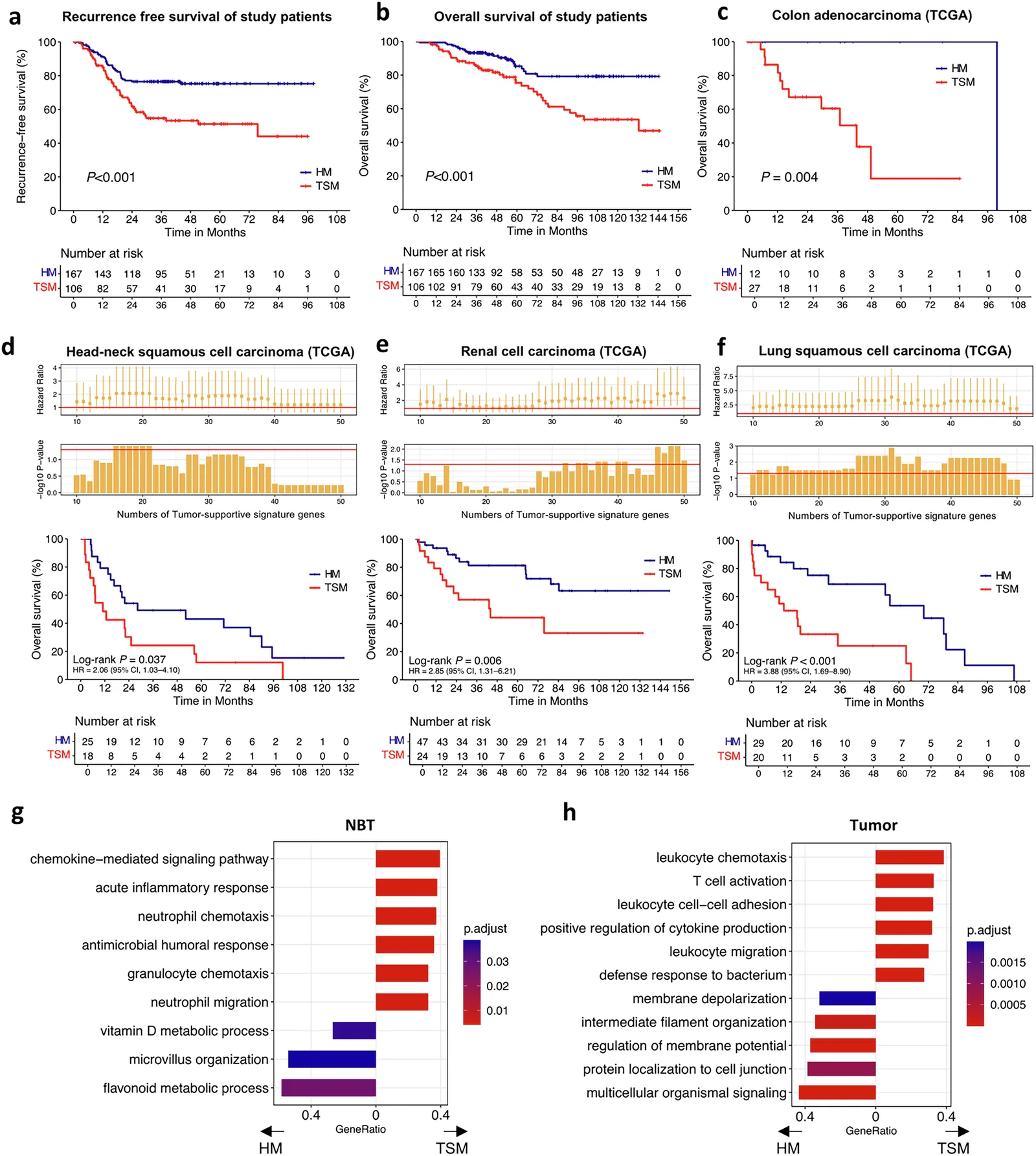
Survival outcomes according to the microenvironment-based classification (TSM versus HM). **a**, **b** RFS (**a**) and OS (**b**) of the study cohort. **c** OS in the TCGA colon cancer dataset. The tumor-supportive signature genes and tumor-supportive signature score cutoff from the study cohort were applied to the TCGA dataset for validation. **d**–**f** Upper bar plots indicate the simulation results of prognostic difference (Cox HR between the two groups (TSM versus. HM)). The groups were divided according to the mean scores of the tumor-supportive signature in the NBT. Tumor-supportive signature gene sets were selected on the basis of the highest HR; when HRs were identical, the set with the fewest genes was chosen. The *x* axis indicates the number of DEGs (mostly overexpressed in tumors) used for calculation, and the *y* axis indicates the HR (top) and −log *P* value (bottom) between the two groups (TSM versus HM). Lower graphs indicate the OS of patients with head and neck squamous cell carcinoma (**d**), renal cell carcinoma (**e**) and lung squamous cell carcinoma (**f**). **g**, **h** GSEA for NBT (**g**) and tumors (**h**). *P* value was adjusted using the Benjamini–Hochberg procedure.

With a median follow-up of 58.2 months, the TSM group showed significantly poorer 5-year RFS (51.4% versus 75.2%, *P* < 0.001) (Fig. 2a) and 5-year OS (75.4% versus 85.3%, *P* < 0.001) (Fig. 2b). The most frequent site of recurrence was the liver (40.5%), followed by the lungs (34.5%); however, there was no significant difference in the location of recurrence between the TSM and HM groups (Supplementary Table 3). The unfavorable survival outcomes of the TSM group compared with the HM group were consistent across the subgroups (Supplementary Figs. 2 and 3). Multivariate analysis revealed that TSM was an independent negative prognostic factor for RFS (versus HM, hazard ratio (HR) 2.27 (95% confidence interval (CI) 1.47–3.50), *P* < 0.001) and OS (versus HM, HR 2.50 (95% CI 1.46–4.26), *P* < 0.001) (Supplementary Table 4). These trends were also validated in a CRC dataset from TCGA, showing significantly poorer 5-year OS for TSM compared with HM (18.9% versus 100%, *P* = 0.004) (Fig. 2c). Next, we further investigated whether the prognostication potential differed in 12 other cancer types in the TCGA dataset with more than 20 paired NBT and tumors samples (Supplementary Table 5). For each cancer type, patients were classified into TSM and HM groups on the basis of NBT expression profiles of tumor-supportive signature genes (Supplementary Table 6), using the same approach as applied to CRC. Interestingly, we found that patients in the TSM group had significantly worse prognoses compared with those in the HM group for head and neck squamous cell carcinoma (3-year OS, 56.9% versus 81.3%, HR 2.06 (95% CI 1.03–4.10), *P* = 0.037), renal cell carcinoma (3-year OS, 25.0% versus 68.9%, HR 2.85 (95% CI 1.31–6.21), *P* = 0.006) and lung squamous cell carcinoma (3-year OS, 24.2% versus 49.2%, HR 3.87 (95% CI 1.69–8.90), *P* < 0.001) (Fig. 2d–f).

### NBTs of the TSM group present a decrease in the microvillus maintenance pathway and enrichment of leukocyte chemotaxis signaling

Comparison of immune cell abundance in the tissues inferred by CIBERSORTx^27^ showed significantly higher proportions of monocytes (*P* = 0.003), activated DCs (*P* < 0.001), activated mast cells (*P* = 0.003) and neutrophils (*P* < 0.001), whereas CD8^+^ T cells were less frequent in the NBT of the TSM group compared with the NBT of the HM group (*P* = 0.003) (Supplementary Fig. 4). However, no significant differences in proportions were observed in the tumors between the TSM and HM groups (Supplementary Fig. 5).

Pathway analysis of the NBT of the TSM group exhibited enrichment in signals related to neutrophil chemotaxis and antimicrobial responses. By contrast, the maintenance pathway of microvillus organization and the vitamin and flavonoid metabolic pathways were significantly decreased in the NBT of the TSM group (Fig. 2g). The vitamin and flavonoid metabolic process pathways were previously reported to be correlated with the modulation of colon mucosal barrier permeability, regulation of the intestinal immune system and positively shaped microbiota^58,59^. Meanwhile, the tumors in the TSM groups also revealed a relative decrease in the epithelial maintenance pathway, alongside enrichment in leukocyte chemotaxis signals and bacterial responses (Fig. 2h).

**Fig. 3:**
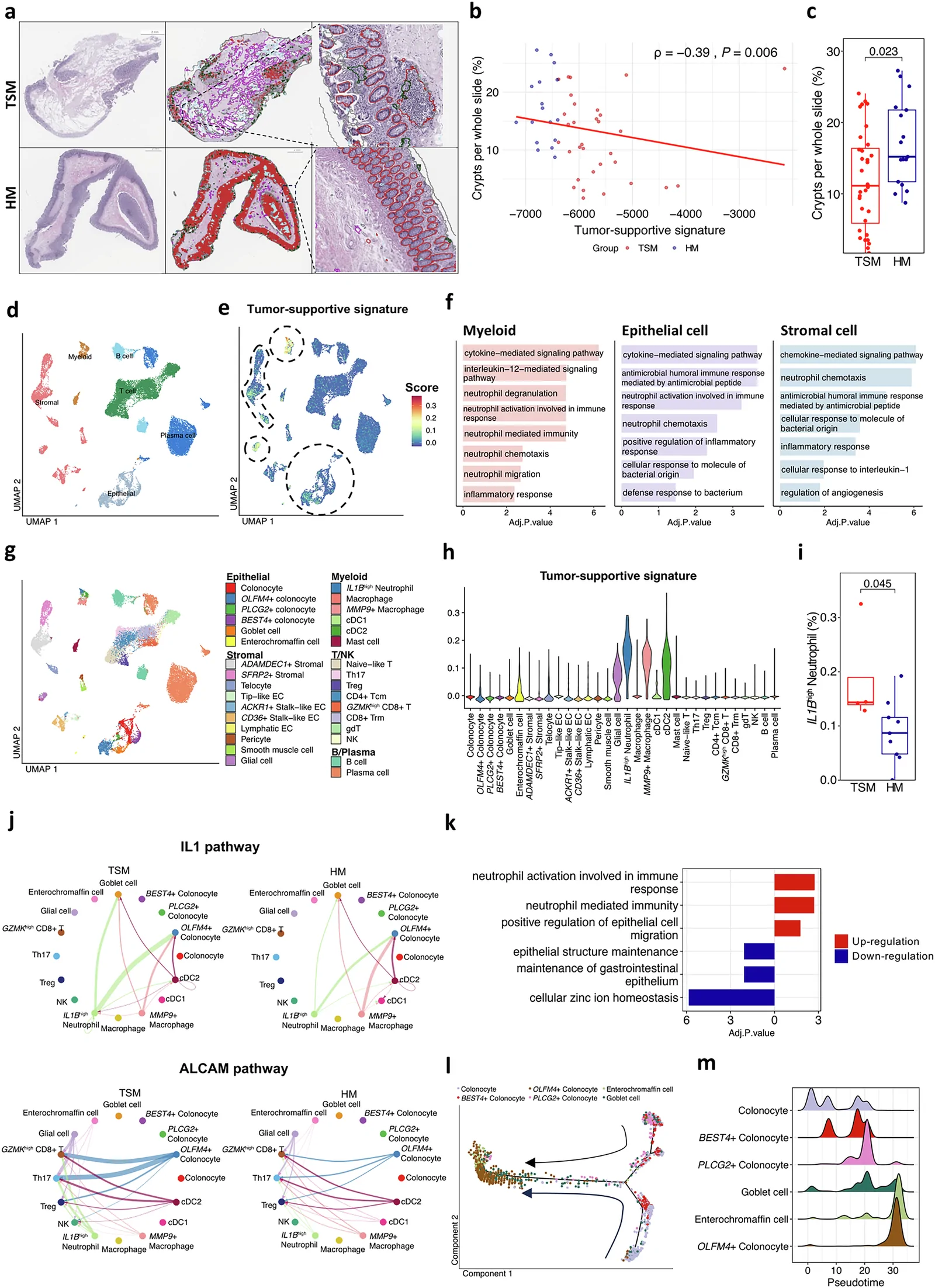
Comprehensive analysis of NBT. **a** H&E-stained slides and AI-powered whole-slide image analysis of the NBT. Red boundary line on the right highlights the AI-powered segmentation of the crypts. Figure shows a representative case of the TSM group, showing disordered crypts (top) and a case from the HM group, showing relatively ordered crypts (bottom). **b** Scatter plots depicting the correlations between tumor-supportive signatures (*x* axis) and the proportion of the total crypt area to the total area of the whole-slide image (*y* axis). Red lines represent the linear fit of the data points, with Spearman correlation coefficients (*ρ*) and *P* values indicated. Blue and red dots indicate the TSM and HM groups, respectively. **c** Comparison of crypts per whole slide (%) between the NBT of the TSM and HM groups. **d** UMAP embedding of epithelial, stromal, myeloid, T/NK, B and plasma cells. **e** UMAP presenting the score of a tumor-supportive signature. **f** Enriched pathways in the TSM group compared with the HM group across myeloid cells, epithelial cells and stromal cells. **g** UMAP showing the identified subsets of all immune and nonimmune cells. **h** Violin plot showing tumor-supportive signatures among cell subtypes in NBT. **i**, Box plot showing a comparison of the proportion of *IL1B*^high^ neutrophils between the TSM and HM groups. Statistical analysis was conducted using the Mann–Whitney U test. **j** Interaction analysis of the IL1 and ALCAM pathways between immune and epithelial cells. The thickness of the lines represents the degree of interaction. **k** Enrichment pathway analysis of *OLFM4*^+^ colonocytes compared with other epithelial cells on the basis of the Gene Ontology database. **l** Pseudotime trajectory for epithelial cells in a two-dimensional state space defined by Monocle2, colored by identified epithelial cell subpopulation. **m** Density plot of epithelial cell pseudotime distribution, illustrating the progression dynamics across epithelial cell subpopulations. UMAP uniform manifold approximation and projection.

To histologically confirm the status of microvillus organization, we conducted hematoxylin and eosin (H&E) staining on available NBT samples from the Samsung Medical Center cohort and performed artificial intelligence (AI)-powered crypt analysis, integrating these stained samples with whole-slide images from the TCGA dataset (*n* = 39). In the TSM group, H&E slides displayed relatively disordered crypts, whereas most NBT samples in the HM group exhibited more ordered crypts (Fig. 3a and Supplementary Fig. 6). AI-powered analysis revealed that the crypt area per whole-slide image was negatively correlated with the tumor-supportive signature, showing a significantly lower proportion of crypts per slide in the TSM group (Fig. 3b, c).

### Upregulated interaction between *IL1B*^high^ neutrophils and *OLFM4*^+^ colonocytes in NBTs of the TSM group

We further performed scRNA-seq of the available NBT tissues derived from 12 patients (Fig. 3d–m and Supplementary Fig. 7), among whom 4 patients (33.3%) were classified into the TSM group. A total of 23,521 cells were available for analysis. In the uniform manifold approximation and projection (UMAP) visualization, tumor-supportive signatures appeared more evident in myeloid, epithelial and stromal cells compared with other cells (Fig. 3d,e). Pathway analyses suggested that neutrophil-mediated pathways were elevated in the TSM group in those cell clusters (Fig. 3f). Among the subclusters, the tumor-supportive signatures were elevated in glial cells, *IL1B*^high^ neutrophils, *MMP9*^+^ macrophages and type 2 conventional DC 2 (cDC2) (Fig. 3h). Among these, a higher proportion of *IL1B*^high^ neutrophils was observed in the TSM group compared with the HM group (Fig. 3i). Cell–cell interaction analysis revealed an upregulated IL1 pathway between *IL1B*^high^ neutrophils and *OLFM4*^+^ colonocytes in the TSM group (Fig. 3j). Moreover, the TSM group featured an upregulated pathway between immune cells and *OLFM4*^+^ colonocytes. In particular, the ALCAM pathway, which is known to mediate tissue repair, homeostasis and responses to injury or inflammation, was enriched between *OLFM4*^+^ colonocytes, *GZMK*^high^ CD8^+^ T cells and T helper 17 cells (Th17) in the TSM group (Fig. 3j).

Notably, the *OLFM4*^+^ colonocytes showed an enriched neutrophil-mediated immune response pathway, whereas the epithelial structural response and zinc ion homeostasis pathways were decreased (Fig. 3k). The trajectory analysis revealed that *OLFM4*^+^ colonocytes were enriched in the late stage of development (Fig. 3l, m). These findings are consistent with previous reports showing that *OLFM4*^+^ colonocytes have important roles in colorectal carcinogenesis and related inflammation^60^, suggesting an important role of *OLFM4*^+^ colonocytes in the TSM group.

### Enrichment of *EMP1*^high^ epithelial cells and *IL1B*^high^ neutrophils in the tumors of the TSM group

**Fig. 4:**
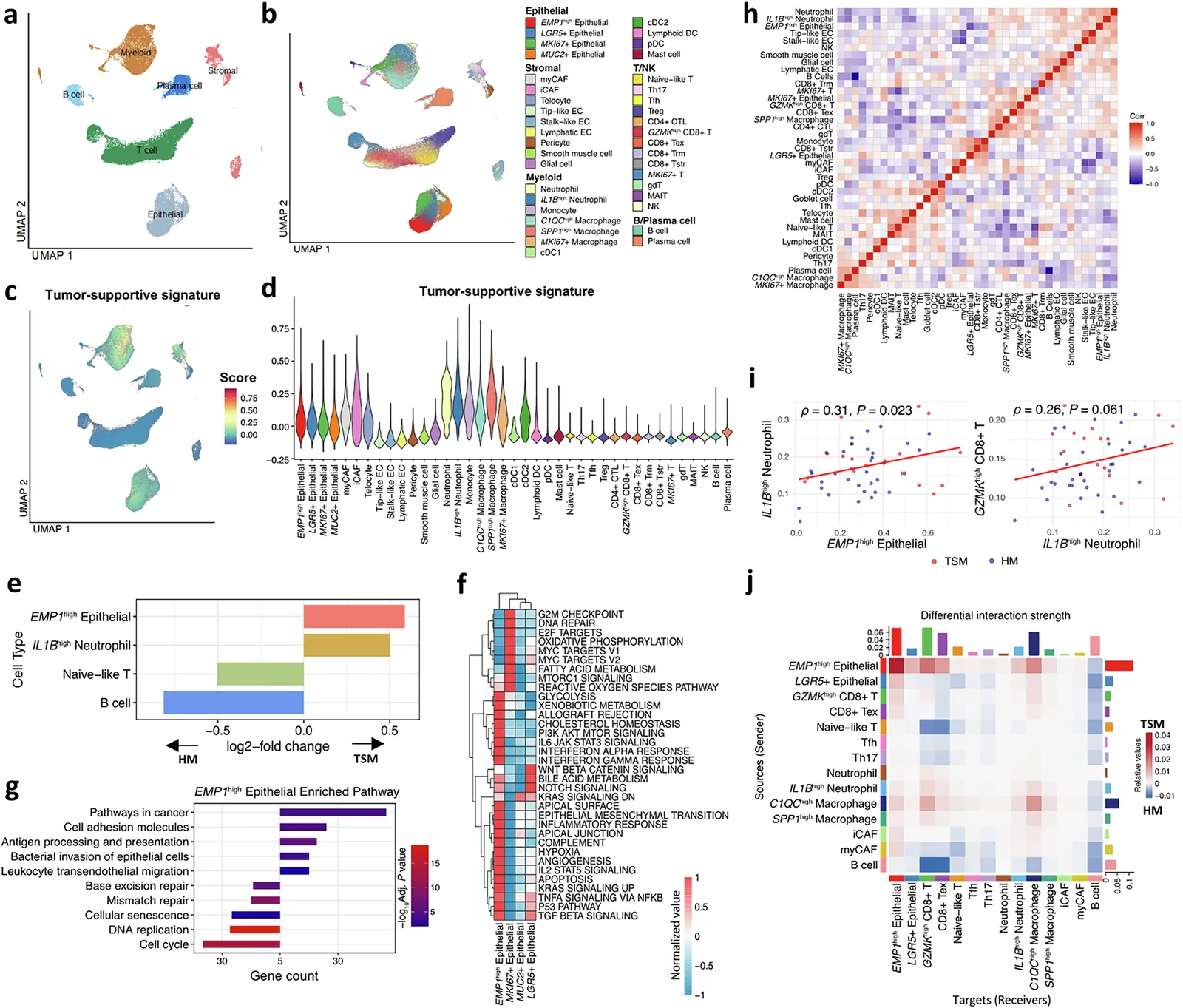
Comprehensive analysis of tumors. **a** UMAP embedding of epithelial stromal, myeloid, T/NK, B and plasma cells. **b** UMAP showing the identified subsets of all immune and nonimmune cells. **c** UMAP presenting the score of a tumor-supportive signature. **d** Violin plot showing tumor-supportive signatures among cell subtypes in tumors. **e** Comparison analysis of cell composition between the TSM and HM groups using scCODA. **f** Heat map illustrating the expression of MSigDB hallmark gene sets across epithelial cell clusters. **g** Enrichment pathway analysis of *EMP1*^high^ epithelial cells compared with other epithelial cells based on the KEGG pathway database. **h**, **i** Correlation analysis of cell proportions among different cell types. Panel h shows a Pearson correlation heatmap, and panel i shows scatter plots with linear fit lines. Red lines represent the linear fit of the data points, with Pearson correlation coefficients (*ρ*) and *P* values indicated. **j** Heat map of interaction scores of ligands and receptors between selected epithelial cells, fibroblasts, myeloid cells and T cells. Red color on the heat map indicates upregulated interactions between cell clusters in the TSM group. Blue color suggests upregulated interactions in the HM group. The color scale reflects the relative strength of ligand–receptor signaling interactions between cell types, as inferred by CellChat.

To further evaluate the cellular features of the tumors in the TSM group, we conducted scRNA-seq for the 52 available tumor tissues derived from 47 patients (Fig. 1h and Supplementary Table 7), among whom 17 patients (36.2%) were classified into the TSM group. In addition, four patients experienced recurrence—three had metachronous recurrence (one involving the liver, one with peritoneal seeding and one with lung recurrence) and one had local recurrence. A total of 118,718 cells were available for scRNA-seq analysis (Fig. 4 and Supplementary Fig. 8). We conducted a cell composition comparison analysis between the TSM and HM groups using scCODA^33^ (Fig. 4e). The TSM group exhibited a higher abundance of the *EMP1*^high^ epithelial cell cluster and neutrophils expressing high levels of *IL-1B* (*IL1B*^high^ neutrophils), which are reported to play a pivotal role in the initiation and orchestration of inflammation, and innate and adaptive immunity, while also being associated with carcinogenesis and metastasis^61^. However, the proportions of naive-like T and B cells, which were previously reported to have an association with a favorable prognosis in CRCs^62,63^, were significantly lower compared with those in the HM group. The *EMP1*^high^ epithelial cells displayed high levels of epithelial–mesenchymal transition (EMT), inflammatory response and angiogenesis pathways in the Molecular Signatures Database (MSigDB) (Fig. 4f). In the Kyoto Encyclopedia of Genes and Genomes (KEGG) pathway database, the *EMP1*^high^ epithelial cell cluster exhibited an upregulation of pathways related to cancer, bacterial invasion and leukocyte migration. By contrast, the cell cycle and DNA damage repair pathways were downregulated (Fig. 4g). These characteristics are in line with previously reported features of *Fusobacterium nucleatum*-infected cancer cells^8^, which exhibit heightened expression of neutrophil chemoattractant chemokines and activation of cancer-related signaling pathways, including extracellular matrix remodeling, metastasis, cell adhesion and migration. In addition, these cells showed downregulated pathways associated with cell cycle regulation and DNA damage repair.

### *EMP1*^high^ epithelial cells, *IL1B*^high^ neutrophils and *GZMK*^high^ CD8^+^ T cells interact more in the tumors of the TSM

**Fig. 5:**
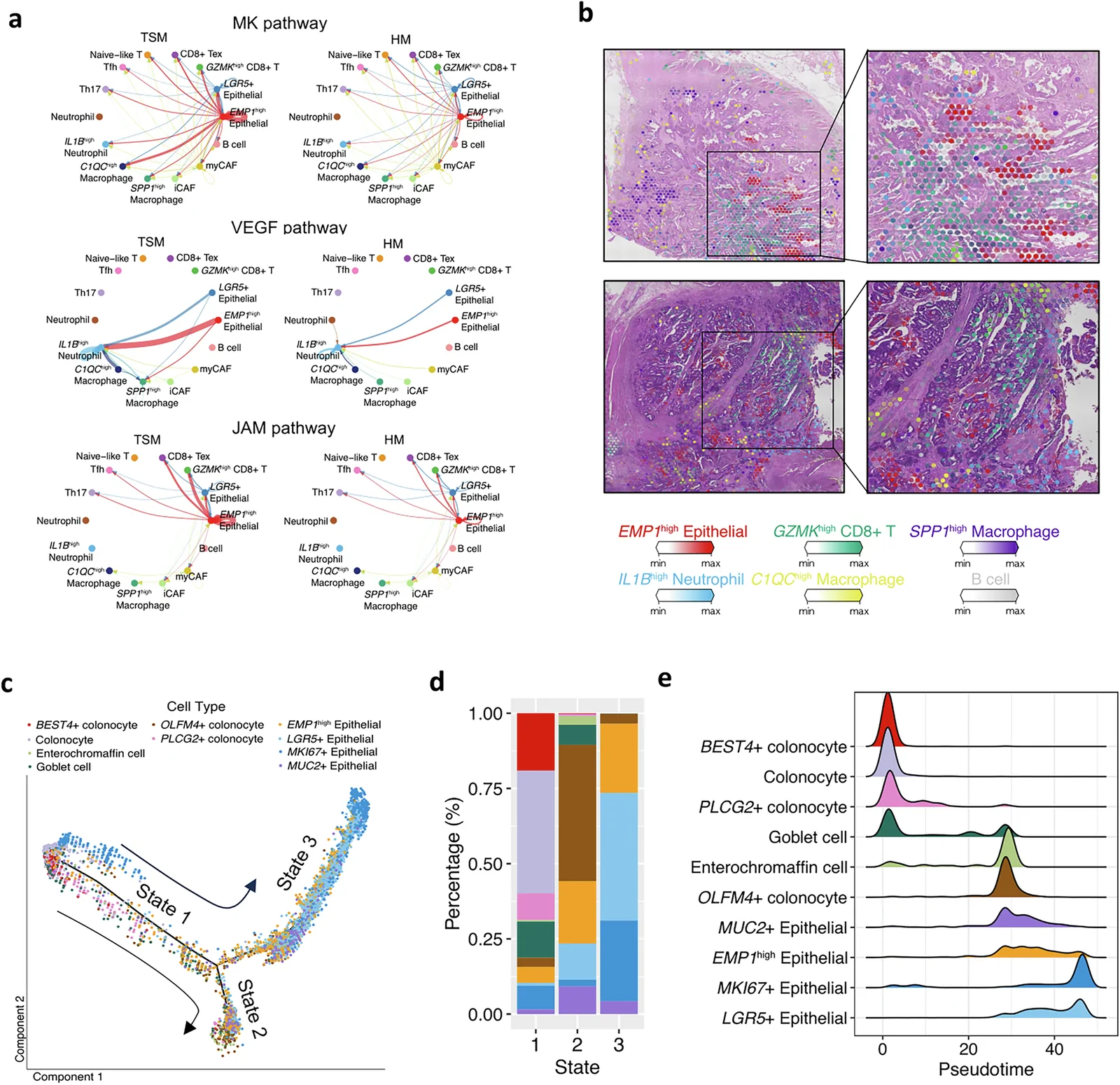
Pathway and spatial transcriptomic analyses. **a** Network analysis of the MK, VEGF and JAM pathways between CAFs and myeloid, epithelial and CD8^+^ T cells. In the network, each node corresponds to a signaling pathway. Edge colors denote the originating sender cell types, while edge thickness reflects the interaction strength predicted by CellChat, with thicker lines indicating stronger signaling. **b** Colocalizations of *EMP1*^high^ epithelial cells, *IL1B*^high^ neutrophils and *GZMK*^high^ CD8^+^ T cells are presented. Data were generated using the 10x Genomics Visium platform (GSE226997). The cell2location tool was used. In public samples 1 (top) and 2 (bottom), colocalization of *EMP1*^high^ epithelial cells with *IL1B*^high^ neutrophils and *GZMK*^high^ CD8^+^ T cells was observed. **c** Pseudotime trajectory for epithelial cells in a two-dimensional state space defined by Monocle2, colored by identified epithelial cell subpopulation. The trajectory analysis was conducted using NBT and paired tumor samples. **d** Proportional bar plot depicting the distribution of epithelial cell subclusters categorized by pseudotime status. **e** Density plot of epithelial cell pseudotime distribution, illustrating the progression dynamics across epithelial cell subpopulations. CAFs, cancer-associated fibroblasts; MSigDB, Molecular Signatures Database; MK, midkine; VEGF, vascular endothelial growth factor; JAM, junctional adhesion molecules.

Analysis of scRNA-seq data revealed a positive correlation between *EMP1*^high^ epithelial cells and *IL1B*^high^ neutrophils (*ρ* = 0.31, *P* = 0.023), whereas other myeloid cells showed weaker associations. In addition, *GZMK*^high^ CD8^+^ T cells exhibited a marginally positive trend with *IL1B*^high^ neutrophils (*ρ* = 0.26, *P* = 0.061) (Fig. 4h,i). Cell–cell interaction analyses revealed upregulated interactions between *EMP1*^high^ epithelial cells, *GZMK*^high^ CD8^+^ T cells, *IL1B*^high^ neutrophils, *C1QC*^high^ macrophages and *SPP1*^high^ macrophages in the TSM group (Fig. 4j). The most prominent pathway in the tumors of the TSM group was the midkine (MK) pathway (Fig. 5a), which promotes EMT, cancer invasion and metastasis through a combination of mitogenic, pro-inflammatory and angiogenic functions^64^. In addition, the vascular endothelial growth factor (VEGF) pathway^65,66^, which is a key signaling pathway that mediates angiogenesis and promotes EMT, was consistently elevated in *EMP1*^high^ epithelial cells and *IL1B*^high^ neutrophils (Fig. 5a). Furthermore, the junctional adhesion molecule (JAM) pathway, which regulates intestinal epithelial proliferation and modulates immune cell interactions and trafficking, playing a role in inflammation^67,68^, was enriched in the TSM group (Fig. 5a).

We further evaluated the spatial distribution of the epithelial and immune cells within the tumor using spatial transcriptomics data (GSE226997)^69^. The cell2location^50^ method was used to map the aforementioned cell types onto the spatial locations of CRC tumors (Fig. 5b and Supplementary Fig. 9). We identified the colocalization of *EMP1*^high^ epithelial cells, *IL1B*^high^ neutrophils and *GZMK*^high^ CD8^+^ T cells, indicating the presence of organized microniches involving immune and epithelial cells.

### Developmental transition of epithelial cells: from NBT to tumor progression

Using NBT and tumor single-cell data, trajectory analysis identified three distinct states, characterized by a bifurcation from state 1 into states 2 and 3 (Fig. 5c). State 1 was predominantly composed of NBT cells, whereas state 3 consisted mainly of tumor cells. State 2 exhibited a mixed proportion of NBT and tumor cells, potentially representing a regenerative or pretumoral state. Notably, *OLFM4*^+^ colonocytes, which play a critical role in the NBT of TSM, were highly abundant, and *MUC2*^+^ epithelial cells were also enriched in this state (Fig. 5d). The *EMP1*^high^ and *LGR5*^+^ epithelial cells were broadly distributed within states 2 and 3, and *MKI67*^+^ epithelial cells were abundant in state 3 (Fig. 5d). Compared with NBT, tumor-related epithelial cells displayed a relatively later stage of development (Fig. 5e). *MUC2*^+^ epithelial cells were observed at an earlier developmental stage in tumors, paralleling the late developmental stage of NBT. *EMP1*^high^ epithelial cells were broadly distributed across both early and late developmental stages of tumors, indicating their persistence and potential role in tumor progression. *LGR5*^+^ epithelial and *MKI67*^+^ proliferative cells were predominantly concentrated in later stages of tumor development, marking a shift toward a more stem-like and actively dividing tumor cell population. This developmental progression profile aligns with findings from a previous study on CRC liver metastasis^40^, where *EMP1*^high^ cells extravasate as clusters, colonizing distant organs as oligocellular structures. In later phases, the reacquisition of *LGR5*^+^ stem cell identity and activation of proliferation programs become critical for tumor outgrowth^70^.

### Microbiota composition in NBTs: similar to tumors in TSM, but distinct in HM

**Fig. 6:**
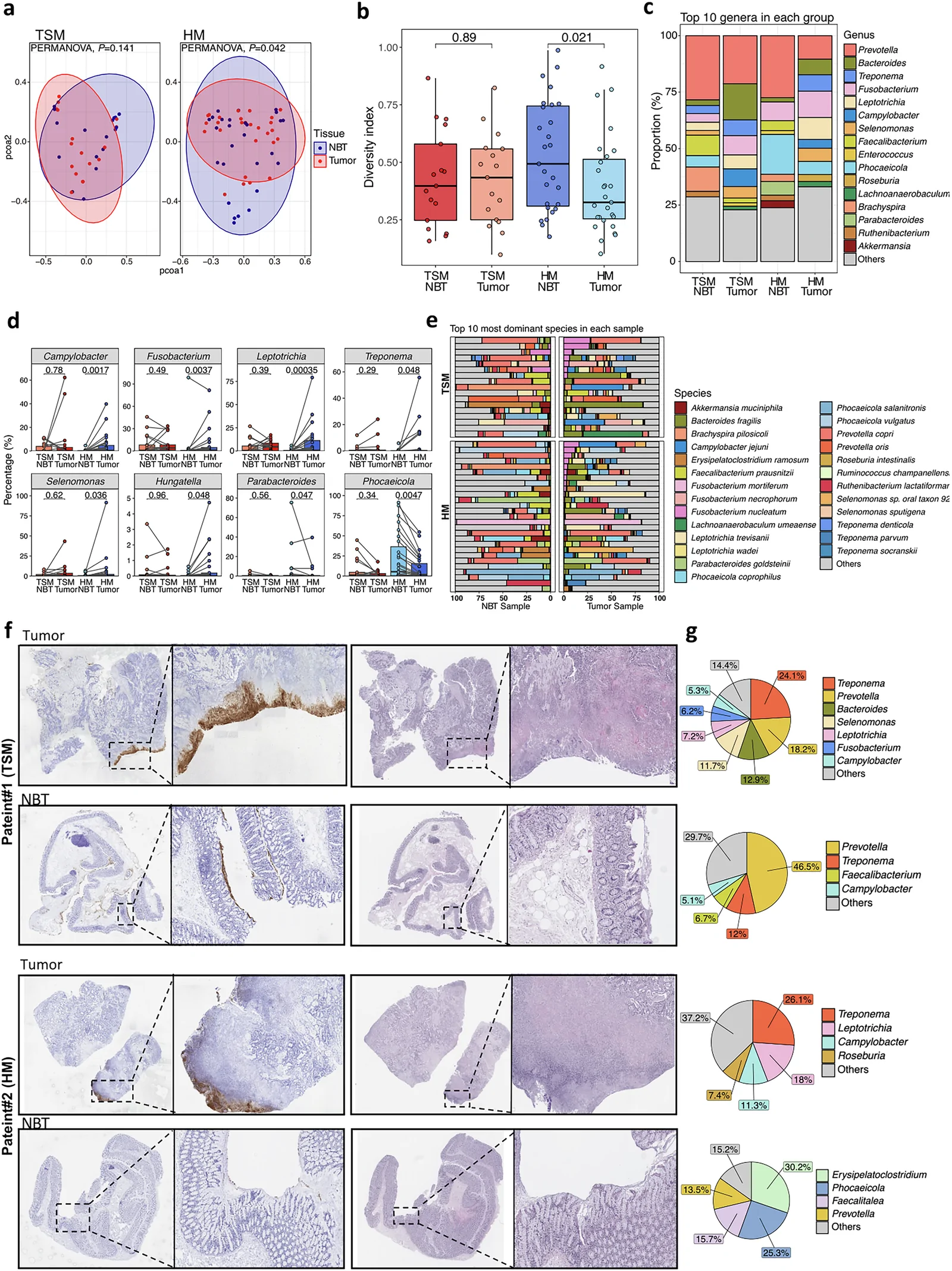
16S rRNA-seq and spatial distribution of eubacteria. **a** Principal coordinate analysis (PCOA) plot representing the beta diversity (Bray–Curtis dissimilarity) of bacterial communities at the genus level from NBT and tumors of the TSM and HM groups, as well as PERMANOVA analyses. **b** Alpha diversity was calculated using the Berger–Parker index. The comparison between the NBT and tumor alpha diversity within the subgroups (TSM and HM) was analyzed using the Wilcoxon signed-rank test. **c** Proportion bar illustrating the relative abundance of bacterial genera according to NBT and tumors of the TSM and HM groups. The relative abundance indicates the total read count of a specific genus per read count from the total genera. **d** Wilcoxon signed-rank test of abundance of bacterial genera between NBT and tumor samples of the TSM and HM groups. Dots indicate the read count of a specific genus divided by the total genera read counts. The *P* values were adjusted using the Benjamini–Hochberg procedure. **e** Individual proportion bar plots of the species based on 16S rRNA-seq. PERMANOVA, permutational multivariate analysis of variance. **f** **g** Representative cases of TSM (patient 1) and HM (patient 2). **f** Images on the left present the results of RNAscope in situ hybridization, indicating the spatial distribution of eubacteria across the tumors and NBT from the surgical specimen (brown color indicates eubacterial probe), with higher magnification (×8) of the bacterial colonization area. Images on the right present H&E-stained slides with higher magnification (×8) of the lesion with inflammatory cell infiltration. **g** Pie charts showing the composition of the bacterial genera derived from the 16S rRNA-seq data. The genera accounting for less than 5% are represented as ‘others’.

Upon observing the enrichment of antimicrobial response pathways in the TSM group through pathway analysis, we conducted 16S rRNA-seq to explore whether a classification scheme based on similarity degree could also reflect differences in the microbiome of the microenvironment. Among the used samples, 17 NBT and 17 tumor samples were derived from the TSM group (*n* = 17, 38.6%) (Supplementary Table 8). We conducted a principal coordinate analysis with beta diversity (Bray–Curtis dissimilarity) of the bacterial community to assess the microbiota resemblance between NBTs and tumors in the TSM and HM groups. No significant differences in the bacterial community were observed between NBT and tumors in the TSM group (PERMANOVA, *F* = 1.288, *P* = 0.141), whereas significant differences were observed in the HM group (PERMANOVA, *F* = 1.563, *P* = 0.042) (Fig. 6a). In the HM group, the alpha diversity (Berger–Parker index) of bacterial communities was significantly higher in the NBT compared with the tumors (Wilcoxon signed-rank test, *P* = 0.021), whereas no significant difference was observed between the NBT and tumors of the TSM group (*P* = 0.890) (Fig. 6b). These results suggest that the microbiome composition in the TSM group was relatively similar between NBT and tumors. By contrast, the HM group exhibited a notable difference in microbiome composition between NBT and tumors, with a more diverse microbiome observed in the NBT. From a genus-level perspective, *Prevotella, Bacteroides, Treponema, Fusobacterium, Leptotrichia, Campylobacter* and *Selenomonas* were the most dominant genera in the tumors of the TSM and HM groups, whereas the proportions of these genera varied between the NBT of the two groups (Fig. 6c).

Next, we conducted a comparison analysis of the proportional abundance of genera between tumors and NBT according to the microenvironment-based subgroups (TSM and HM). Significantly lower proportions of carcinogenic or enterotoxigenic genera (*Campylobacter*^71^*, Fusobacterium*^72–74^*, Leptotrichia*^75^*, Treponema*^76^*, Selenomonas*^75,77^ and *Hungatella*^78^) were observed in the NBT compared with tumors in the HM group. More *Parabacteroides* genera, which inhibit *TLR4* suppression and have antitumor effects^79,80^, and more *Phocaeicola* were detected in the NBT than in tumors in the HM group (Fig. 6d and Supplementary Fig. 10a). By contrast, no significant differences in genus proportions between NBT and tumors were observed in the TSM group. *Prevotella copri* was the most prevalent species in NBT as well as in tumors from both the TSM and HM groups (Fig. 6e and Supplementary Fig. 10b). *Bacteroides fragilis* and *Campylobacter jejuni* were identified as the dominant species in the tumors, particularly in those from the TSM group. *F. nucleatum* was detected at levels above 2% in tumors in 7 out of 17 patients in the TSM group (41.2%) and in 9 out of 27 patients in the HM group (33.3%). In addition, *F. nucleatum* was found exclusively in the NBT of patients in the TSM group (*n* = 3, 17.6%) (Fig. 6e and Supplementary Fig. 10b, c).

We conducted RNAscope in situ hybridization on available paired NBT and tumor samples subjected to 16S rRNA-seq to evaluate the spatial distribution of the microbiota (Fig. 6f, g). Patient 1 was classified into the TSM group, and 16S rRNA-seq revealed *Treponema* and *Prevotella* genera-dominant microbiota in the NBT and paired tumor. Bacterial colonies (brown) were observed in the tumor and NBT mucosal areas (Fig. 6f, g). Patient 2 was classified into the HM group. Bacterial aggregation was uniquely observed in the tumor, whereas it was scarcely present in the NBT. The 16S rRNA-seq of the tumor revealed a *Treponema* genus-dominant microbiota, similar to that of patient 2, whereas 16S rRNA-seq of NBT showed different genera, including symbiotic bacteria such as *Erysipelatoclostridium* and *Phocaeicola* (Fig. 6f, g).

### Putative mechanism

**Fig. 7:**
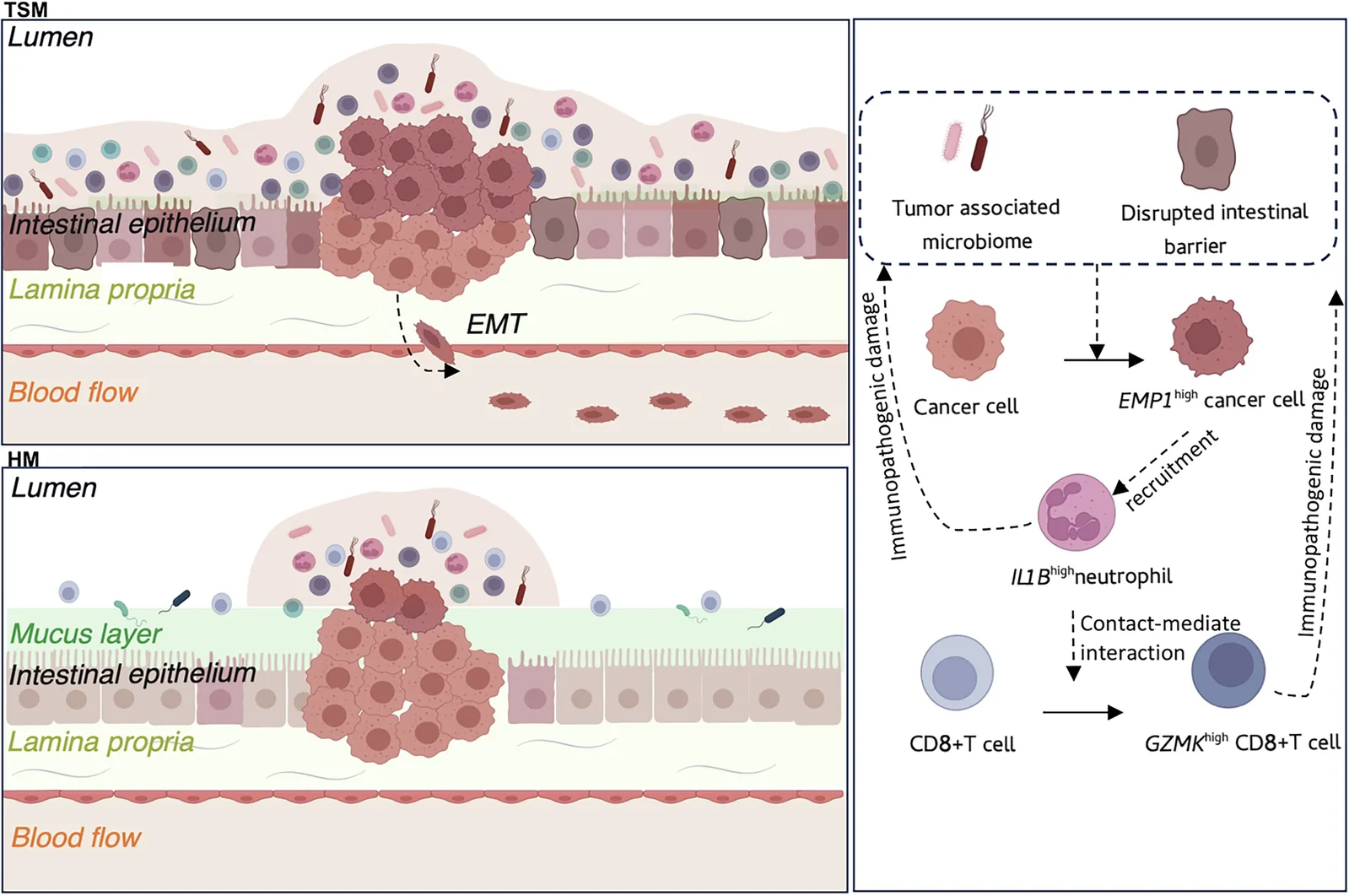
Putative mechanism. Illustrations of the putative mechanisms for the TSM and HM groups. The illustration on the right indicates the putative biological mechanism underlying the vicious cycle in the TSM group.

Overall, the NBT of the TSM group exhibited a tumor-favorable microbiome composition, with an activated bacterial humoral response and decreased intestinal barrier maintenance, highlighting the prominent interaction between *IL1B*^high^ neutrophils and *OLFM4*^+^ colonocytes. Figure 7 shows the putative mechanism involving organized microniches of immune and epithelial cells revealed by our findings.

The tumors of the TSM group comprised a higher proportion of *EMP1*^high^ epithelial cells, which were related to bacterial invasion, leukocyte signaling and the EMT pathway. Those epithelial cell clusters may provoke *IL1B*^high^ neutrophil retention. Subsequently, these neutrophils crosstalk with CD8^+^ T cells, inducing the production of high levels of *GZMK*, which in turn decreases E-cadherin in the intestinal epithelium and promotes tumor progression^40^. A weak intestinal barrier may provoke more invasion of bacteria into the cancer cells, promoting cancer cell progression via EMT and recruiting *IL1B*^high^ neutrophils and *GZMK*^high^ CD8^+^ T cells, resulting in a vicious cycle. These organized microniches, comprising immune and epithelial cells, may contribute to cancer recurrence after surgical resection.

Within the NBT of the TSM group, *IL1B*^high^ neutrophils may promote epithelial plasticity and *OLFM4* expression in colonocytes through chronic inflammatory signaling. Complementarily, *OLFM4*⁺ colonocytes, reflecting a regenerative or stress-adapted phenotype^60,81,82^, may facilitate further neutrophil recruitment or retention. This mutual interaction probably contributes to a self-perpetuating inflammatory loop that fosters a TSM. By analyzing the bulk-level transcriptomes of NBT, we were able to classify the subgroups with distinct prognostic outcomes that exhibited varying levels of microniche abundance within the tumors.

## Discussion

This study presents a different perspective by showing that NBT, traditionally considered a control, may reflect a TSM associated with poor prognosis. By contrast with conventional tumor-centric approaches, we stratified patients into TSM and HM groups on the basis of the transcriptomic similarity between tumors and matched NBTs, using tumor-supportive signature genes. The TSM group exhibited significantly worse outcomes, and their NBTs showed features of epithelial barrier disruption, neutrophil-driven inflammation and tumor-like microbiome composition. Unlike previous studies that primarily described molecular features of NBTs without providing prognostic validation or mechanistic explanation, our study demonstrates the clinical relevance and mechanistic basis of NBT-based classification by leveraging multi-center clinical data with a multi-omics approach.

We failed to discern significant differences in the specific immune cell proportions within the tumors between the two groups using a bulk-level RNA-seq-based deconvolutional approach. However, by applying scRNA-seq analysis, we identified a greater proportion of *EMP1*^high^ epithelial cells interacting with *IL1B*^high^ neutrophils and *GZMK*^high^ CD8^+^ T cells in the tumors of the TSM group compared with the HM group. This result may also support our hypothesis, which underscores the importance of examining not only the tumor but also the NBT because bulk-level RNA-seq analysis for tumors is inevitably influenced by intratumoral heterogeneity. By focusing on the NBT, we were able to discern two groups with distinct microniche abundance in the tumor and different prognoses. This was also supported by a bulk RNA-seq dataset from TCGA, which included colorectal and other cancers, highlighting the potential prognostic role of NBT as a biomarker.

The NBT of the TSM group exhibited tumor-supportive signature scores similar to those observed in colon polyps (*P* = 0.690), whereas the NBT of the HM group showed significantly lower scores (*P* = 0.017). Given that CRC is often preceded by a polypoid precursor^83^, these findings may support our hypothesis that the more closely the NBT resembles the tumor, the higher the likelihood of recurrence. In addition, the colorectal microenvironment of patients with Crohn’s disease showed significantly higher tumor-supportive signatures compared with the NBTs of our study cohort, which did not have a history of inflammatory bowel disease (*P* < 0.001). If patients with CRC and Crohn’s disease were enrolled, they would probably be classified under the TSM group, aligning with the fact that the CRC arising in patients with Crohn’s disease is associated with poor outcomes^84^.

Given the poor prognosis of the TSM subgroup, additional management following surgical resection is warranted. Our pathway analysis highlighted that the NBT of the TSM group showed decreased maintenance of epithelial integrity and flavonoid or vitamin metabolic process pathway activity. In addition, our 16S rRNA-seq analysis indicated that the NBT of the TSM group had a relatively similar bacterial community to that of tumors, suggesting the presence of a more tumor-favorable microbiome in this subgroup. Flavonoid and vitamin metabolic pathways influence the colonic mucosa and positively shape the microbiota^58^. Consistently, a recent randomized controlled trial showed that a high flavonoid intake had clinical benefits in patients who underwent surgical resection for CRC^85^ and a post hoc analysis from a prospective cohort showed that higher predicted vitamin D status was associated with significantly reduced recurrence and improved survival in resected colon cancer^86^. These findings suggest that dietary interventions may hold promise for improving outcomes in the TSM group. Furthermore, emerging microbiome-modulatory strategies, such as probiotics or fecal microbiota transplantation, have shown potential to reshape the gut microbial and immune landscape^87–90^. Whether such approaches can reprogram the tumor-supportive microbiota observed in the TSM group into a more favorable postsurgical environment warrants further investigation.

In addition, the TSM group featured higher proportions of *EMP1*^high^ epithelial cells. A recent human-like mouse model-based study investigated the mechanisms underlying metachronous recurrence after surgical resection of CRC. This study reported that the *EMP1*^high^ epithelial cell cluster was enriched in liver micrometastases with high T cell infiltration and became progressively immune-excluded during outgrowth^70^. These results suggest a potential role for perioperative immunotherapy in CRC. As the TSM group features a high proportion of the *EMP1*^high^ epithelial cell cluster, the group’s prognosis may be prolonged by adjuvant immunotherapy after surgery. Importantly, the clinical utility of adjuvant immunotherapy is currently under investigation (NCT02912559 and NCT03827044). Thus, once the findings of these trials are revealed, the potential benefits to the TSM subgroup require further evaluation.

Furthermore, *IL1B*^high^ neutrophils appear to play a central role in both compartments: they are enriched in tumors harboring *EMP1*^high^ epithelial cells associated with bacterial invasion and EMT signaling, as well as in NBTs containing *OLFM4*⁺ colonocytes that exhibit a stress-adapted epithelial phenotype. These coordinated interactions suggest that interleukin (IL)-1β-mediated neutrophil activity may orchestrate a tumor-supportive inflammatory environment^91^ spanning both tumor and NBT. IL-1β-targeted therapies are currently undergoing clinical evaluation across a range of inflammatory and oncologic diseases^92–94^. Whether perioperative modulation of the IL-1β axis can reduce recurrence or improve outcomes in resected CRC remains to be determined, particularly in high-risk subgroups such as those with the TSM phenotype.

Our study had some limitations. First, its retrospective design presents unintentional biases. Second, although our classification was externally validated using the TCGA cohort, its reliance on a cohort-specific cutoff may not fully account for underlying biological heterogeneity and could limit generalizability to other datasets. Third, although the framework demonstrated prognostic utility across multiple cancer types, the use of cancer-specific gene sets limits generalizability. Fourth, we were unable to capture the functionality of the microbiome and its associated metabolomic aspects, warranting further prospective validation studies.

In conclusion, we could discern patients with a poor prognosis by evaluating the status of the colorectal microenvironment. This group exhibited a weakened intestinal barrier and a tumor-promoting bacterial community within the colorectal microenvironment. These findings highlight the importance of maintaining a healthy colorectal microenvironment, potentially through dietary interventions or additional treatments.

## Supplementary information

Supplementary Information

## Data Availability

The bulk RNA sequencing data generated in this study are available via Zenodo at https://doi.org/10.5281/zenodo.8170823 (ref. ^95^), https://doi.org/10.5281/zenodo.8170863 (ref. ^96^), https://doi.org/10.5281/zenodo.8170850 (ref. ^97^) and https://doi.org/10.5281/zenodo.8170960 (ref. ^98^). The raw 16S rRNA sequencing data are available in the Sequence Read Archive under BioProject ID no. PRJNA743150. Additional data and materials used in this study are available upon reasonable request and subject to review for potential conflicts with existing intellectual property rights or confidentiality obligations.

## Code availability

A subset of the scripts used for bulk RNA-seq, scRNA-seq and microbiome analysis workflows—particularly those related to tumor-supportive signature scoring and downstream visualizations—are available via GitHub at https://github.com/SGI-pan/NBT_code. Additional code and custom pipelines are available from the corresponding author upon reasonable request.
