# Supplementary material for: Colorectal microenvironment determines the prognosis of colorectal cancer
Source: Exp Mol Med. 2026 Jan 7;58(1):110–25. doi: 10.1038/s12276-025-01599-7 (PMC12868731; doi:10.1038/s12276-025-01599-7)
Supplement: Supplementary file 1 — Supplementary Information [file 12276_2025_1599_MOESM1_ESM.pdf]

Supplementary materials

Colorectal Microenvironment Determines the Prognosis of Colorectal Cancer

Y.H. Bang & J.H. Choi *et al.*

Supplementary Figures

Supplementary Fig. 1: Supplementary materials for RNA sequencing

Supplementary Fig. 2: Comparison of prognostic outcomes according to cohort characteristics

Supplementary Fig. 3: Forest plot of hazard ratios for recurrence-free survival and overall survival according to TSM and HM

Supplementary Fig. 4: Composition of immune cells in the NBT calculated by the CIBERSORTx algorithm

Supplementary Fig. 5: Composition of immune cells in the tumor calculated by the CIBERSORTx algorithm

Supplementary Fig. 6: H&E-stained slides of the NBT

Supplementary Fig. 7: Expression of canonical markers across cell subtypes within NBT

Supplementary Fig. 8: Supplementary materials for single-cell RNA sequencing analysis of tumors

Supplementary Fig. 9: Co-localization analysis among different cell types with scRNA-seq data and spatial Visium data

Supplementary Fig. 10: Supplementary materials for 16S rRNA sequencing

Supplementary Tables

Supplementary Table 1: Differentially expressed genes between non-tumor-bearing tissues (NBT) and tumors used for scoring the tumor-supportive signature

Supplementary Table 2: Comparison of baseline characteristics between patients with tumor-supportive microenvironment (TSM) and patients with healthy microenvironment (HM) (*n* = 273)

Supplementary Table 3: Comparison of recurrence site between TSM and HM groups

Supplementary Table 4: Multivariate analyses for recurrence-free survival and overall survival

Supplementary Table 5: Number of NBT–tumor pairs across different cancer types for tumor-supportive signature scoring in the TCGA dataset

Supplementary Table 6: Tumor-supportive signature genes in various cancer types (TCGA dataset)

Supplementary Table 7: Baseline characteristics comparison between TSM and HM groups with single-cell RNA sequencing analysis (*n* = 47)

Supplementary Table 8: Baseline characteristics comparison between TSM and HM groups with 16S rRNA sequencing analysis (*n* = 44)

SUPPLEMENTARY FIGURES

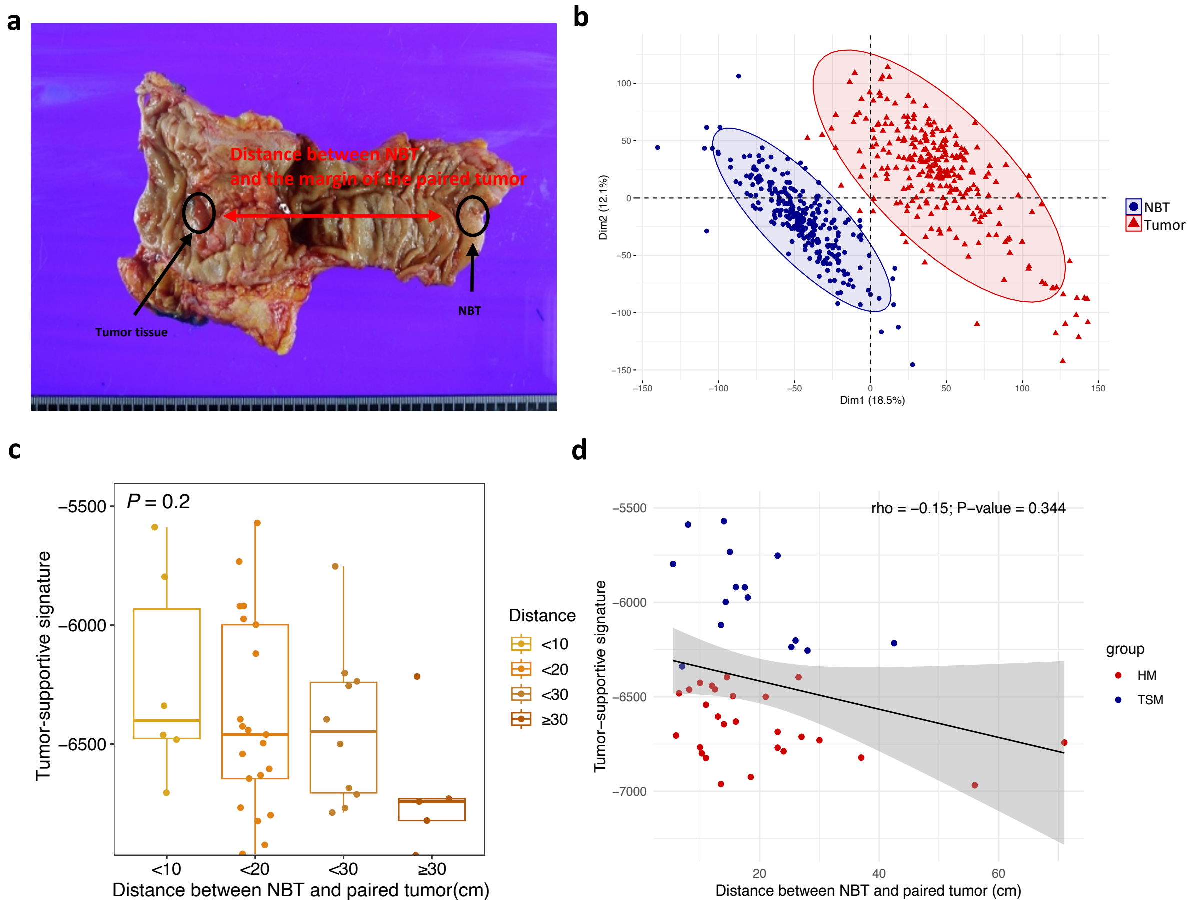

Supplementary Fig. 1: Supplementary materials for RNA sequencing. (a) Sample acquisition sites, and distance between NBT and the margin of the tumor. **(b)** Principal component analysis results of RNA sequencing of NBT and tumor samples. **(c)** Comparison of tumor-supportive signature scores based on the distance between paired NBT and tumor margin. **(d)** Scatter plot showing the correlation between tumor-supportive signature score and NBT-tumor distance

NBT: non-tumor-bearing tissue

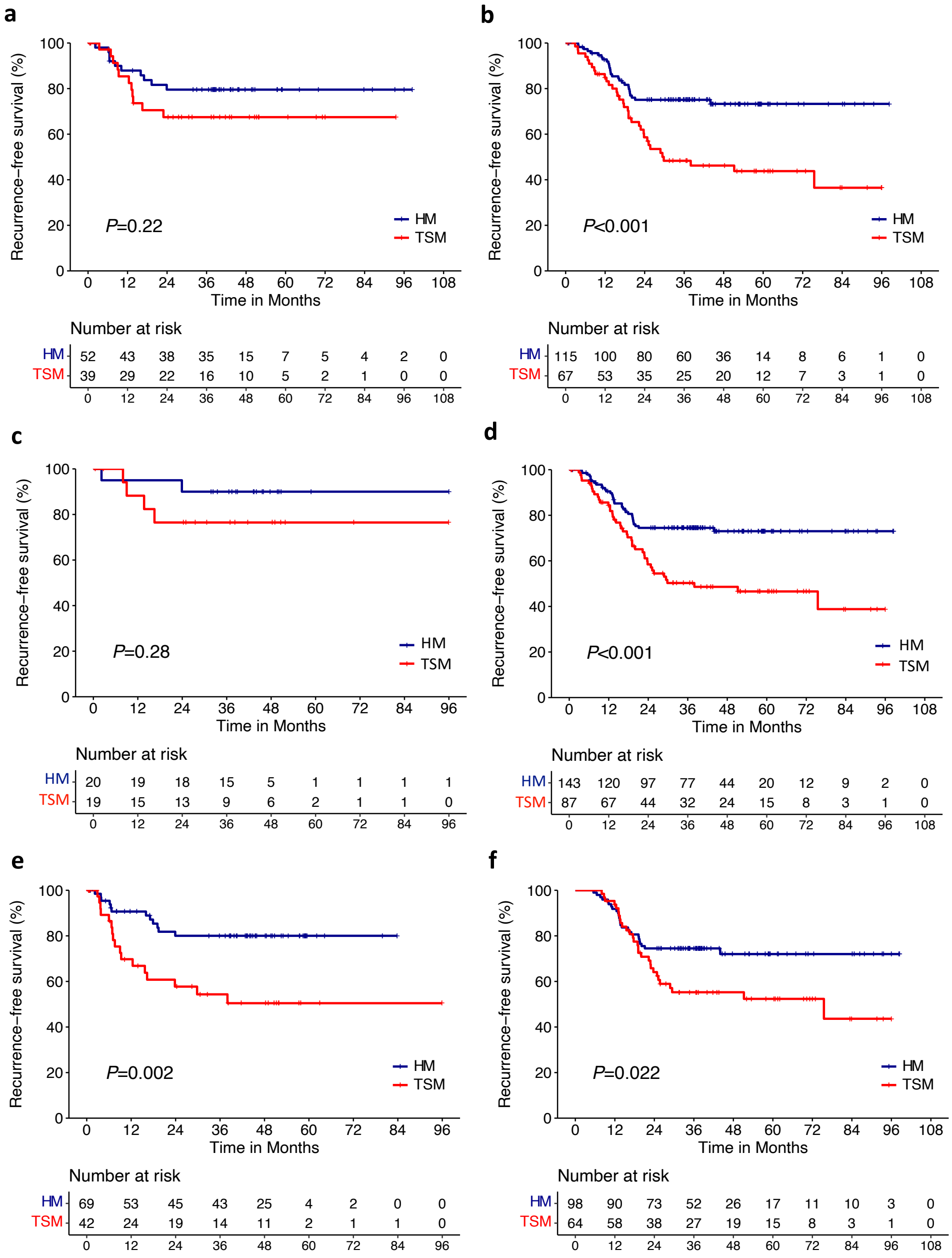

Supplementary Fig. 2: Comparison of prognostic outcomes according to cohort characteristics. (a, b) Recurrence-free survival in right- **(a)** and left-sided CRC **(b)**. **(c, d)** Recurrence-free survival in patients with CRC with MSI-H **(c)**, MSI-L, or MSS **(d)**. **(e, f)** Recurrence-free survival in patients with CRC at the Samsung Medical Center **(e)** and other centers **(f)**.

TSM, tumor-supportive microenvironment; HM, healthy microenvironment; CRC, colorectal cancer; MSI-H, microsatellite instability-high; MSI-L, microsatellite instability-low; MSS, microsatellite stable

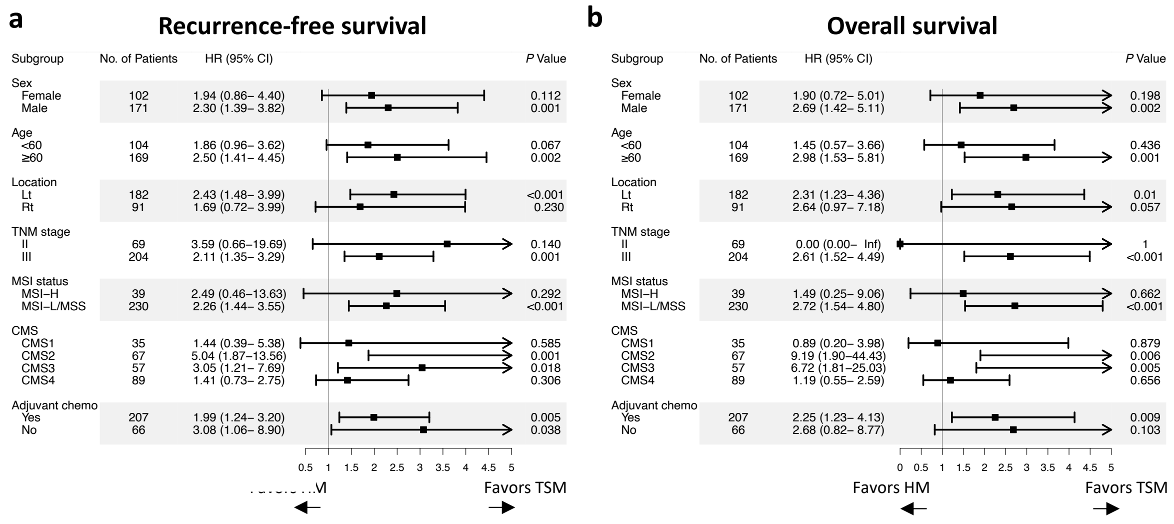

Supplementary Fig. 3: Forest plot of hazard ratios for recurrence-free survival and overall survival according to TSM and HM. Forest plot of the hazard ratio (dot) and 95% CI (arrow) for recurrence-free survival **(a)** and overall survival **(b)** according to TSM and HM.

HR, hazard ratio; Lt, left sided; Rt, right sided; CMS, consensus molecular subtype; chemo, chemotherapy

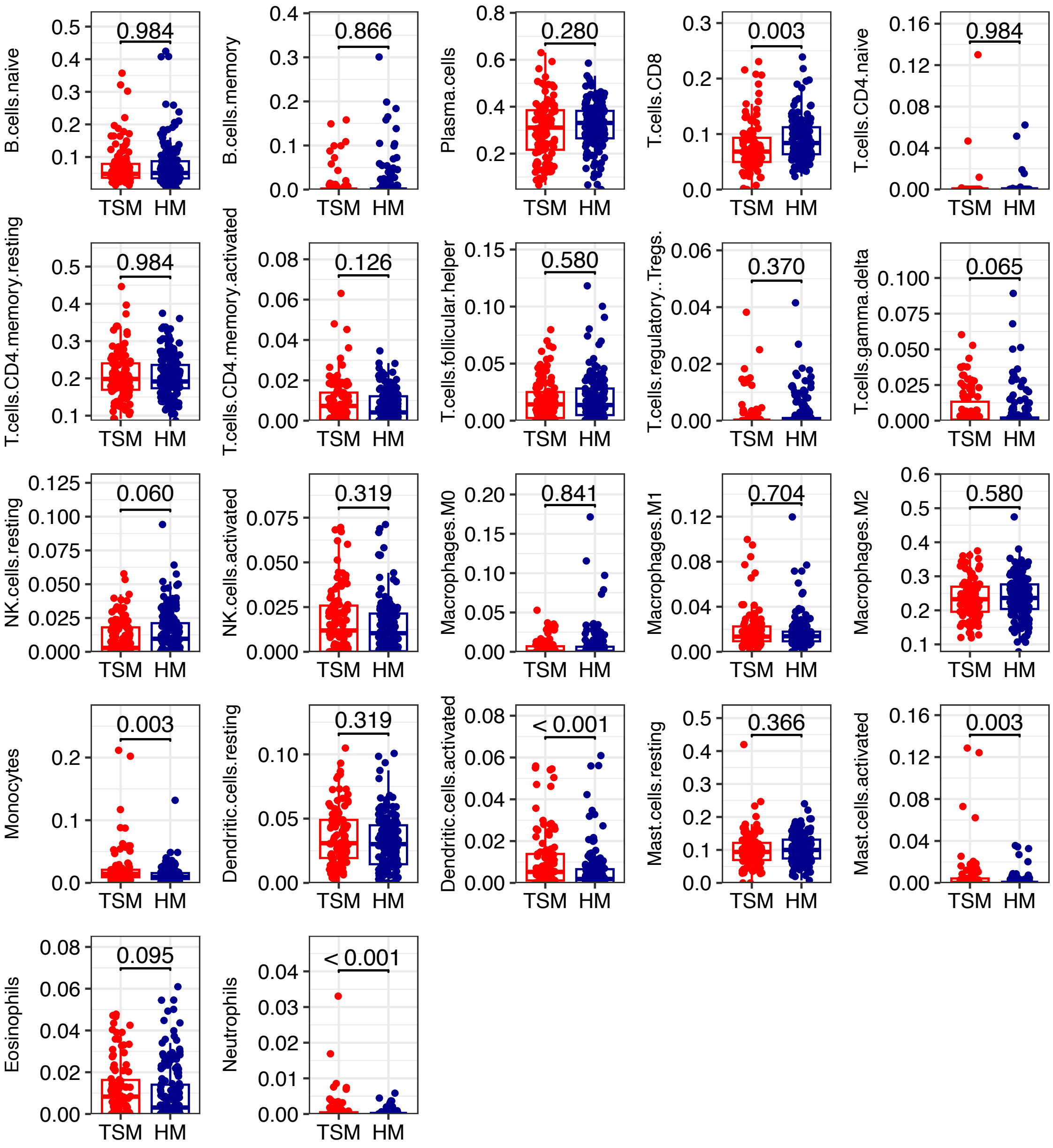

Supplementary Fig. 4: Composition of immune cells in the NBT calculated by the CIBERSORTx algorithm.

P-value was calculated using the Wilcoxon rank-sum test. The *P*-values were adjusted by Benjamini–Hochberg.

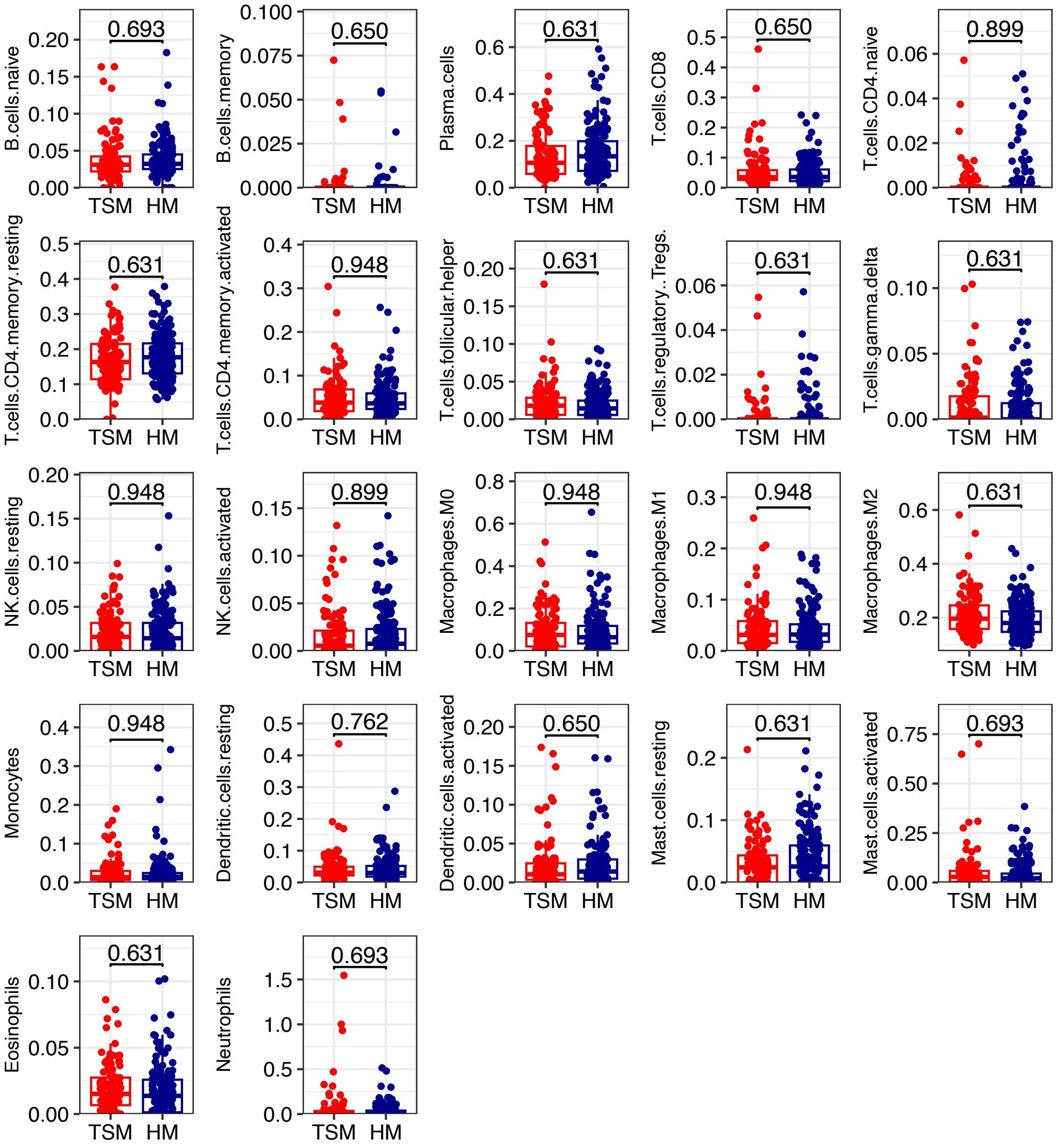

Supplementary Fig. 5: Composition of immune cells in the tumor calculated by the CIBERSORTx algorithm. *P*-value was calculated using the Wilcoxon rank-sum test. The *P*-values were adjusted by Benjamini–Hochberg.

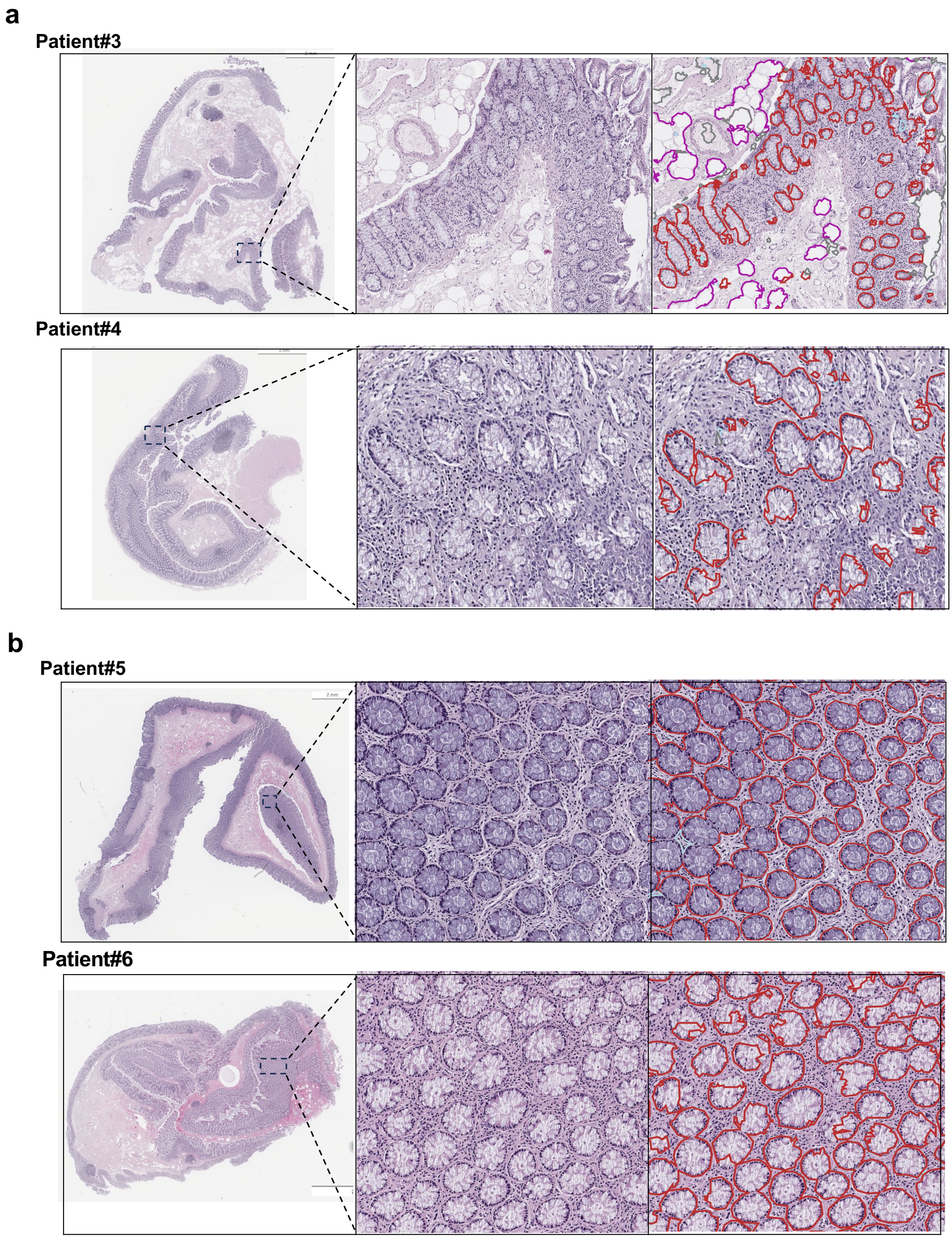

Supplementary Fig. 6: H&E-stained slides of the NBT. (a, b) H&E-stained slides and artificial intelligence-powered whole-slide image analysis of the NBT in the TSM group (Patient#3 and Patient#4) **(a)** and HM group (Patient#5 and Patient#6) **(b)**. The red boundary line on the right highlights the AI-powered segmentation of the crypts.

H&E: Hematoxylin and eosin

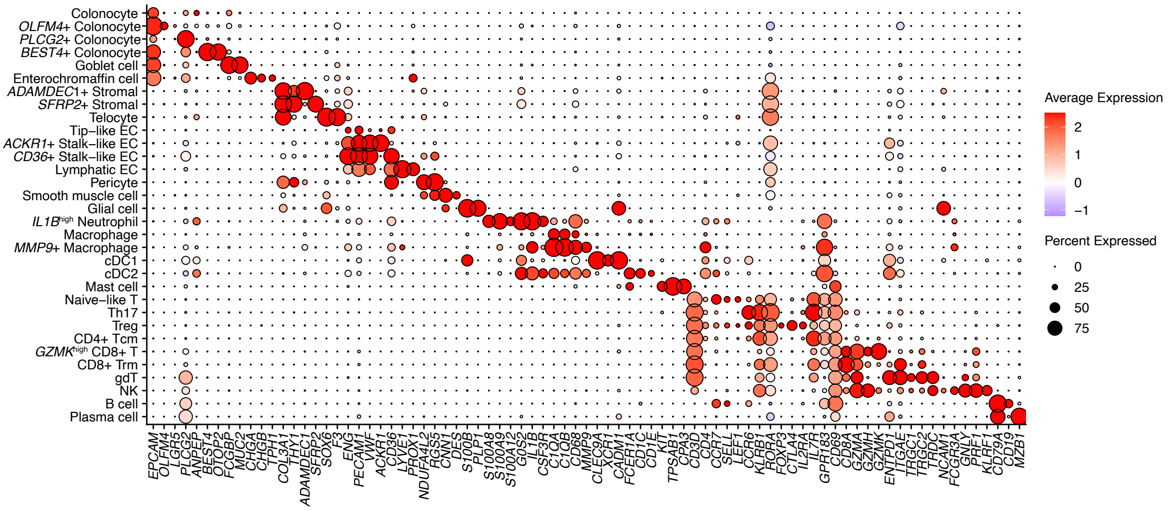

Supplementary Fig. 7: Expression of canonical markers across cell subtypes within NBT.

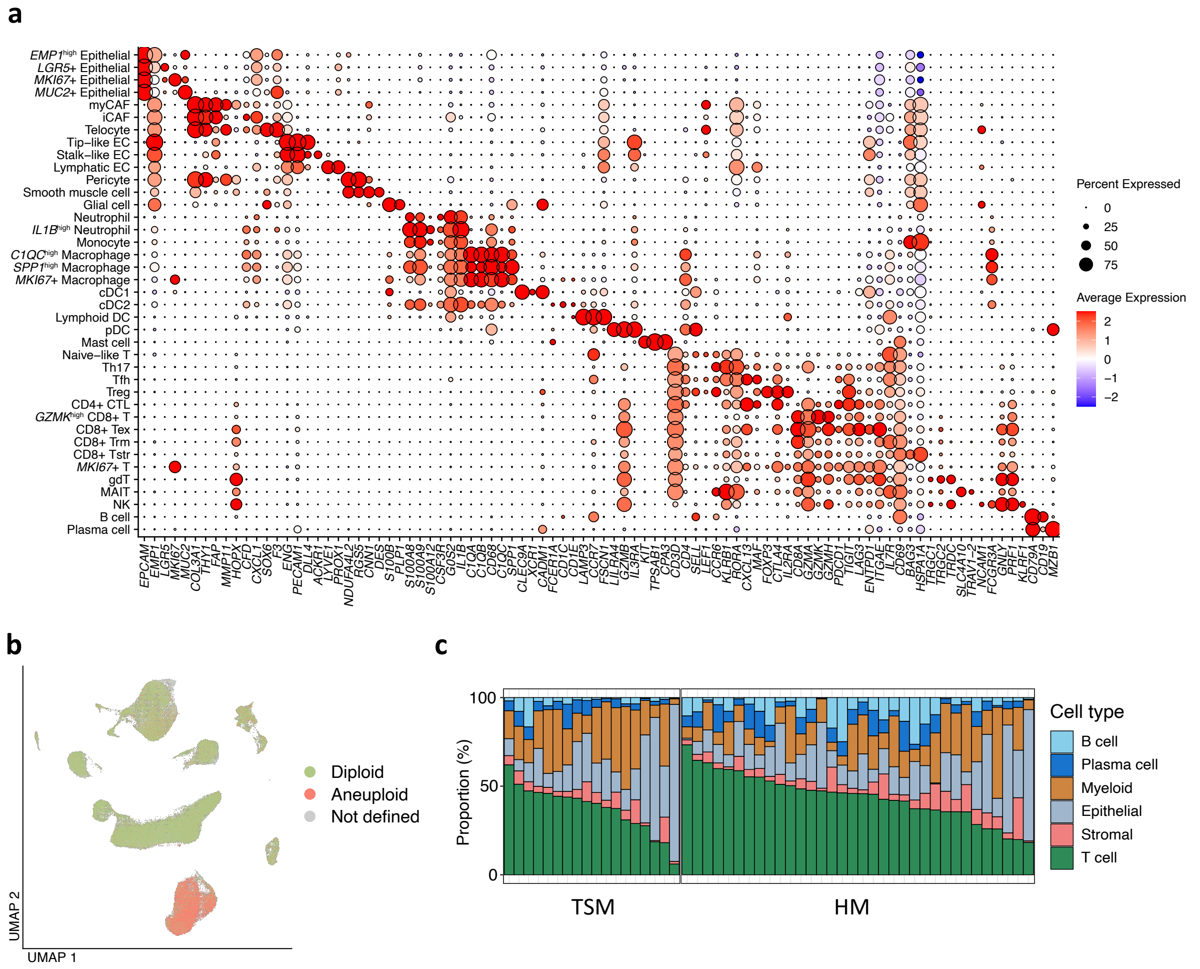

Supplementary Fig. 8: Supplementary materials for single-cell RNA sequencing analysis of tumors. (a) Dot plot showing the expression of canonical markers across cell subtypes within tumors. **(b)** UMAP plot indicates the distribution of aneuploid and diploid cells in the global cells of tumor samples. Most aneuploid cells were epithelial cell clusters. **(c)** Proportion bar plot illustrating the global distribution of cell types.

UMAP: Uniform manifold approximation and projection

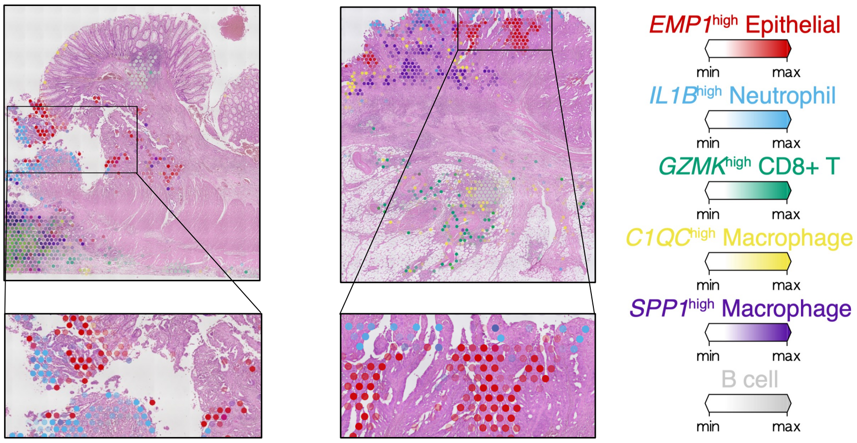

Supplementary Fig. 9: Co-localization analysis among different cell types with scRNA-seq data and spatial Visium data. Visium data from publicly available CRC dataset (GSE226997) show the localization of *EMP1*^{high} epithelial cells, *IL1B*^{high} neutrophils, *GZMK*^{high} CD8^{+} T cells, *C1QC*^{high} macrophages, *SPP1*^{high} macrophages, and B cells. The cell2location tool was used to map these cell types onto the spatial locations of the tumor. In samples 3 (left) and 4 (right), co-localization of *EMP1*^{high} epithelial cells with *IL1B*^{high} neutrophils was observed.

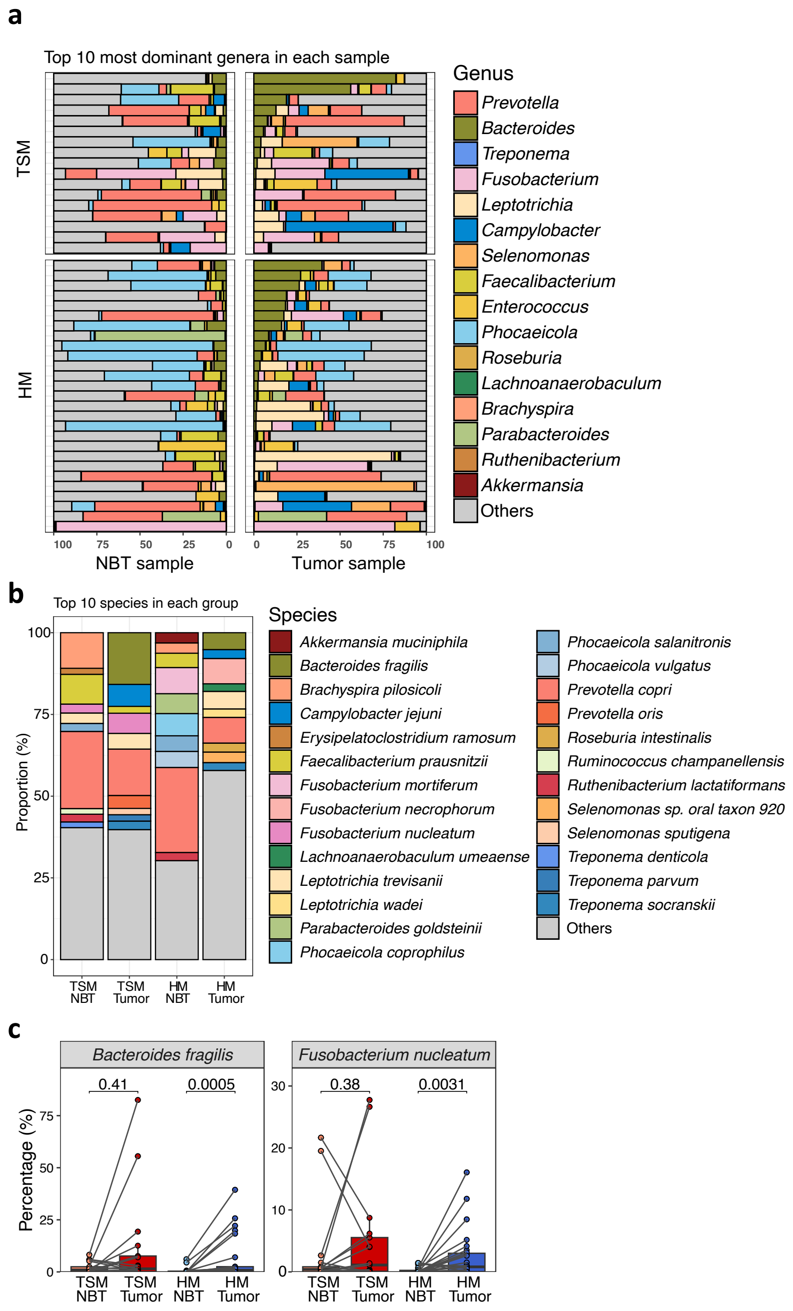

Supplementary Fig. 10: Supplementary materials for 16S rRNA sequencing. (a) Individual proportion bar plots of the genera based on 16S rRNA sequencing. The relative abundance indicates the total read count of a specific genus per read count from the total genera. (**b)** Proportion bar illustrating the relative abundance of bacterial species according to NBT and tumors of the TSM and HM groups. The relative abundance indicates the total read count of a specific species per read count from the total species. (**c)** Wilcoxon signed-rank test of abundance of bacterial species between NBT and tumor samples of the TSM and HM groups. The *P*-values were adjusted by Benjamini–Hochberg. Dot indicates the read count of a specific species divided by the total species read counts.

Supplementary Table 1: Differentially expressed genes between non-tumor-bearing tissues (NBT) and tumors used for scoring the tumor-supportive signature

Function	Gene symbol	Gene name
Maintaining epithelial barrier integrity	*CDH3*	cadherin 3
	CLDN2	claudin 2
	COL10A1	collagen type X alpha 1 chain
	DSG3	desmoglein 3
	KRT17	keratin 17
	KRT23	keratin 23
	KRT6B	keratin 6B
	REG1B	regenerating family member 1 beta
	REG3A	regenerating family member 3 alpha
Neutrophil chemotaxis	*CXCL5*	C-X-C motif chemokine ligand 5
	CXCL8	C-X-C motif chemokine ligand 8
	PPBP	pro-platelet basic protein
	SPP1	secreted phosphoprotein 1
Growth factors	*FOXQ1*	forkhead box Q1
	INHBA	inhibin subunit beta A
	NOTUM	notum, palmitoleoyl-protein carboxylesterase
	WNT2	Wnt family member 2
Extracellular matrix components	*MMP3*	matrix metallopeptidase 3
	MMP7	matrix metallopeptidase 7
Cellular environment regulation	*CA9*	carbonic anhydrase 9
	SLCO1B3	solute carrier organic anion transporter family member 1B3
	TCN1	transcobalamin 1
Others	*COL11A1*	collagen type XI alpha 1 chain
	CST1	cystatin SN
	DPEP1	dipeptidase 1
	HCAR3	hydroxycarboxylic acid receptor 3
	MTTP	microsomal triglyceride transfer protein
	S100A2	S100 calcium binding protein A2

Note: Overexpressed genes in tumors (with high fold-change values) were designated as tumor-supportive signature genes.

Supplementary Table 2: Comparison of baseline characteristics between patients with tumor-supportive microenvironment (TSM) and patients with healthy microenvironment (HM) (*n* = 273)

	TSM (*n* = 106)	HM (*n* = 167)	*P*-value
**Age**			0.133
≥60 years	72 (67.9%)	97 (58.1%)	
<60 years	34 (32.1%)	70 (41.9%)	
**Sex**			0.999
Male	66 (62.3%)	105 (62.9%)	
Female	40 (37.7%)	62 (37.1%)	
**Tumor site side**			0.404
Right sided	39 (36.8%)	52 (31.1%)	
Left sided	67 (63.2%)	115 (68.9%)	
**TNM stage (AJCC 8th edition)**			0.934
Stage II	26 (24.5%)	43 (25.7%)	
Stage III	80 (75.5%)	124 (74.3%)	
**Extended stage**			0.964
T1–3 and N0–1	63 (59.4%)	101 (60.5%)	
T4 and/or N2	43 (40.6%)	66 (39.5%)	
**MSI status**			0.054
MSI-H	19 (17.9%)	20 (12.0%)	
MSI-L	1 (0.9%)	9 (5.4%)	
MSS	86 (81.1%)	134 (80.2%)	
NA	0 (0.0%)	4 (2.4%)	
**CMS**			0.217
CMS 1	17 (16.0%)	18 (10.8%)	
CMS 2	22 (20.8%)	45 (26.9%)	
CMS 3	17 (16.0%)	40 (24.0%)	
CMS 4	40 (37.7%)	49 (29.3%)	
NA	10 (9.4%)	15 (9.0%)	
**Adjuvant chemotherapy**			0.157
Yes	75 (70.8%)	132 (79.0%)	
No	31 (29.2%)	35 (21.0%)	

Data are presented as n (%), unless otherwise indicated. *P*-value was calculated using the chi-square test.

Abbreviations: AJCC, American joint committee on cancer; MSI, microsatellite instability; MSI-H, microsatellite instability-high; MSI-L, microsatellite instability-low; MSS, microsatellite stable; CMS, consensus molecular subtype; NA, not available for analysis.

Supplementary Table 3: Comparison of recurrence site between TSM and HM groups

Site of recurrence	Total<br>(*n* = 84)	TSM<br>(*n* = 46)	HM<br>(*n* = 38)	*P*-value
Liver	34 (40.5%)	19 (43.2%)	15 (39.5%)	0.908
Lung	29 (34.5%)	14 (30.4%)	15 (39.5%)	0.524
Lymph node	15 (17.9%)	8 (18.2%)	7 (18.4%)	0.999
Peritoneum	14 (16.7%)	8 (18.2%)	6 (15.8%)	0.999
Colon	7 (8.3%)	6 (13.6%)	1 (2.6%)	0.116
Others	5 (6.0%)	3 (6.8%)	2 (5.3%)	0.187

Data are presented as numbers (%). *P*-values were calculated using the chi-square test or Fisher's exact test.

Supplementary Table 4: Multivariate analyses for recurrence-free survival and overall survival

Variables	Recurrence-free survival				Overall survival			
	Univariate		Multivariate		Univariate		Multivariate	
	HR (95% CI)	*P*-value	HR (95% CI)	*P*-value	HR (95% CI)	*P*-value	HR (95% CI)	*P*-value
**Age** (≥60 *vs*. <60 years)	0.97 (0.63–1.5)	0.889			1.77 (1.01–3.10)	0.046	1.86 (1.06–3.26)	0.030
**Sex** (Male *vs*. Female)	1.71 (1.06–2.76)	0.029	1.54 (0.94–2.52)	0.083	1.43 (0.81–2.52)	0.218		
**Tumor location** (Rt. sided *vs*. Lt. sided)	0.69 (0.42–1.14)	0.146			0.98 (0.55–1.72)	0.934		
**TNM stage** **(**Stage III *vs*. Stage II)	5.25 (2.29–12.06)	<0.001	4.37 (1.89–10.11)	<0.001	11.77 (1.62–85.65)	0.015	12.66 (1.74–92.19)	0.012
**NBT-based classification** (TSM *vs*. HM)	2.21 (1.43–3.39)	<0.001	2.27 (1.47–3.50)	<0.001	2.46 (1.44–4.19)	<0.001	2.50 (1.46–4.26)	<0.001
**MSI status** (MSI-L or MSS *vs*. MSI-H)	2.42 (1.06–5.57)	0.037	2.19 (0.95–5.05)	0.066	1.35 (0.54–3.38)	0.526		
**Adjuvant chemotherapy** (Yes *vs*. No)	1.08 (0.63–1.87)	0.773			0.60 (0.33–1.11)	0.105		

Abbreviations: HR, hazard ratio; CI, confidence interval; Rt. sided, right-sided; Lt. sided, left-sided; MSI, microsatellite instability; MSI-H, microsatellite instability-high; MSI-L, microsatellite instability-low; MSS, microsatellite stable; TSM, patients with tumor-supportive microenvironment; HM, patients with healthy microenvironment.

Supplementary Table 5: Number of NBT–tumor pairs across different cancer types for tumor-supportive signature scoring in the TCGA dataset

Cancer type	Number of paired NBT and tumor samples (pair)
BRCA	111
HNSC	43
KICH	22
KIRC	71
KIRP	31
LIHC	50
LUAD	57
LUSC	49
PRAD	52
STAD	26
THCA	58
UCEC	23

Only cancer types with 20 or more NBT–tumor pairs were used in the analysis.

Abbreviations: BRCA, breast cancer; HNSC, head and neck squamous cell carcinoma; KICH, kidney chromophobe; KIRC, kidney renal cell carcinoma; KIRP, kidney renal papillary cell carcinoma; LIHC, liver hepatocellular carcinoma; LUAD, lung adenocarcinoma; LUSC, lung squamous cell carcinoma; PRAD, prostate adenocarcinoma; STAD, stomach adenocarcinoma; THCA, thyroid carcinoma; UCEC, uterine corpus endometrial carcinoma; NBT, non-tumor-bearing tissue.

Supplementary Table 6: Tumor-supportive signature genes in various cancer types (TCGA dataset)

HNSC	LUSC		KIRC	
CALB1	*BAAT*	*C5orf46*	*KRT31*	*AKR1B10*
MAGEA4	*MYEOV*	*LGI4*	*KRT14*	*CERS3*
MAGEA3	*C10orf99*	*PNCK*	*CALML3*	*RHCG*
MMP13	*CCL18*	*CHIT1*	*S100A7*	*TMPRSS11D*
COL10A1	*TNFAIP6*	*ANGPTL4*	*KRT6C*	*LY6D*
CA9	*FABP7*	*GABRD*	*IGFL1*	*SPRR3*
PRAME	*MCHR1*	*SAA1*	*S100A7A*	*FOXE1*
MMP11	*CA9*	*COL23A1*	*MAGEA4*	*SPRR2A*
COL11A1	*FABP6*	*SAA2*	*GJB6*	*SERPINB13*
HMGA2	*PTHLH*	*GRIK3*	*KRT16*	*A2ML1*
FBN2	*RAB42*	*CD70*	*DSG3*	*KRTDAP*
MMP9	*SLC6A3*	*ENPP3*	*CALML5*	*SPRR2E*
INHBA	*NDUFA4L2*	*HP*	*AMTN*	*KRT5*
PTHLH	*CYP2J2*	*HILPDA*	*KRT6B*	*SPRR1A*
STC2	*APOC1*	*SCGN*	*TMPRSS11A*	*IL36RN*
LAMC2	*NPTX2*		*KRT6A*	*SPRR2D*
			PRAME	*KRT13*
			MMP13	*TMPRSS11E*
			KRT75	*CST1*
			SPRR1B	*SERPINB5*
			KLK6	*DUSP9*
			FAM83C	*IL36G*
			SPRR2G	*SLC6A15*

Genes overexpressed in tumors (with high fold-change values) were designated as tumor-supportive signature genes.

Abbreviations: HNSC, head and neck squamous cell carcinoma; LUSC, lung squamous cell carcinoma; KIRC, renal cell carcinoma.

Supplementary Table 7: Baseline characteristics comparison between TSM and HM groups with single-cell RNA sequencing analysis (*n* = 47)

	TSM (*n* = 17)	HM (*n* = 30)	*P*-value
**Age**			0.321
≥60 years	14 (82.4%)	20 (66.7%)	
<60 years	3 (17.6%)	10 (33.3%)	
**Sex**			0.581
Male	9 (52.9%)	12 (40.0%)	
Female	8 (47.1%)	18 (60.0%)	
**Tumor site side**			0.558
Right sided	14 (82.4%)	21 (70.0%)	
Left sided	3 (17.6%)	9 (30.0%)	
**TNM stage (AJCC 8th edition)**			0.781
Stage II	7 (41.2%)	15 (50.0%)	
Stage III	10 (58.8%)	15 (50.0%)	
**Extended stage**			0.772
T1–3 and N0–1	11 (64.7%)	22 (73.3%)	
T4 and/or N2	6 (35.3%)	8 (26.7%)	
**MSI status**			0.698
MSI-H	8 (47.1%)	11 (36.7%)	
MSS	9 (52.9%)	19 (63.3%)	
**CMS**			0.381
CMS 1	5 (29.4%)	7 (23.3%)	
CMS 2	1 (5.9%)	9 (30.0%)	
CMS 3	4 (23.5%)	6 (20.0%)	
CMS 4	6 (35.3%)	6 (20.0%)	
NA	1 (5.9%)	2 (6.7%)	
**Adjuvant chemotherapy**			0.999
Yes	9 (52.9%)	17 (56.7%)	
No	8 (47.1%)	13 (43.3%)	

Data are presented as n (%), unless otherwise indicated. *P*-value was calculated using the chi-square test, as appropriate.

Abbreviations: AJCC, American joint committee on cancer; MSI, microsatellite instability; MSI-H, microsatellite instability-high; MSI-L, microsatellite instability-low; MSS, microsatellite stable; CMS, consensus molecular subtype; NA, not available for analysis.

Supplementary Table 8. Baseline characteristics comparison between TSM and HM groups with 16S rRNA sequencing analysis (*n* = 44)

	TSM (*n* = 17)	HM (*n* = 27)	*P*-value
**Age**			0.486
≥60 years	14 (82.4%)	19 (70.4%)	
<60 years	3 (17.6%)	8 (29.6%)	
**Sex**			0.631
Male	9 (52.9%)	11 (40.7%)	
Female	8 (47.1%)	16 (59.3%)	
**Tumor site side**			0.999
Right sided	14 (82.4%)	21 (77.8%)	
Left sided	3 (17.6%)	6 (22.2%)	
**TNM stage (AJCC 8th edition)**			0.704
Stage II	7 (41.2%)	14 (51.9%)	
Stage III	10 (58.8%)	13 (48.1%)	
**Extended stage**			0.952
T1–3 and N0–1	11 (64.7%)	19 (70.4%)	
T4 and/or N2	6 (35.3%)	8 (29.6%)	
**MSI status**			0.921
MSI-H	8 (47.1%)	11 (40.7%)	
MSS	9 (52.9%)	16 (59.3%)	
**CMS**			0.298
CMS 1	5 (29.4%)	7 (25.9%)	
CMS 2	1 (5.9%)	8 (29.6%)	
CMS 3	4 (23.5%)	6 (22.2%)	
CMS 4	6 (35.3%)	4 (14.8%)	
NA	1 (5.9%)	2 (7.4%)	
**Adjuvant chemotherapy**			0.999
Yes	9 (52.9%)	15 (55.6%)	
No	8 (47.1%)	12 (44.4%)	

Data are presented as n (%), unless otherwise indicated. *P*-value was calculated using the chi-square test.

Abbreviations: AJCC, American joint committee on cancer; MSI, microsatellite instability; MSI-H, microsatellite instability-high; MSI-L, microsatellite instability-low; MSS, microsatellite stable; CMS, consensus molecular subtype; NA, not available for analysis.
